# Isolation and Comprehensive Analysis of Cochlear Tissue‐Derived Small Extracellular Vesicles

**DOI:** 10.1002/advs.202408964

**Published:** 2024-11-05

**Authors:** Pei Jiang, Xiangyu Ma, Xinlin Wang, Jingyuan Huang, Yintao Wang, Jingru Ai, Hairong Xiao, Mingchen Dai, Yanqin Lin, Buwei Shao, Xujun Tang, Wei Tong, Zixuan Ye, Renjie Chai, Shasha Zhang

**Affiliations:** ^1^ State Key Laboratory of Digital Medical Engineering Department of Otolaryngology Head and Neck Surgery Zhongda Hospital School of Life Sciences and Technology Advanced Institute for Life and Health Jiangsu Province High‐Tech Key Laboratory for Bio‐Medical Research Southeast University Nanjing 210096 China; ^2^ Southeast University Shenzhen Research Institute Shenzhen 1518063 China; ^3^ School of Medicine Faculty of Medical & Health Sciences Tel Aviv University Tel Aviv 6997801 Israel; ^4^ Department of Otolaryngology Head and Neck Surgery Sichuan Provincial People's Hospital University of Electronic Science and Technology of China Chengdu 610072 China; ^5^ Co‐Innovation Center of Neuroregeneration Nantong University Nantong 226001 China; ^6^ Institute for Stem Cell and Regeneration Chinese Academy of Science Beijing 100101 China

**Keywords:** cochlea, FGFR1, hair cells, miRNA, small extracellular vesicle, supporting cells

## Abstract

Small extracellular vesicles (sEVs) act as a critical mediator in intercellular communication. Compared to sEVs derived from in vitro sources, tissue‐derived sEVs can reflect the in vivo signals released from specific tissues more accurately. Currently, studies on the role of sEVs in the cochlea have relied on studying sEVs from in vitro sources. This study evaluates three cochlear tissue digestion and cochlear tissue‐derived sEV (CDsEV) isolation methods, and first proposes that the optimal approach for isolating CDsEVs using collagenase D and DNase І combined with sucrose density gradient centrifugation. Furthermore, it comprehensively investigates CDsEV contents and cell origins. Small RNA sequencing and proteomics are performed to analyze the miRNAs and proteins of CDsEVs. The miRNAs and proteins of CDsEVs are crucial for maintaining normal auditory function. Among them, FGFR1 in CDsEVs may mediate the survival of cochlear hair cells via sEVs. Finally, the joint analysis of single CDsEV sequencing and single‐cell RNA sequencing data is utilized to trace cellular origins of CDsEVs. The results show that different types of cochlear cells secrete different amounts of CDsEVs, with Kölliker's organ cells and supporting cells secrete the most. The findings are expected to enhance the understanding of CDsEVs in the cochlea.

## Graphical abstract

This study establishes an optimal isolation method for neonatal cochlear tissue‐derived small extracellular vesicles (CDsEVs), and further comprehensively investigates their contents and cell origins by small RNA‐seq, proteomics, and single sEV sequencing. Notably, FGFR1 protein in CDsEVs may mediate the survival of cochlear hair cells via sEVs, suggesting CDsEVs may play roles in intracellular communication in cochlea.

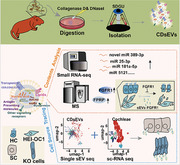

## 1 Introduction

Extracellular vesicles (EVs) belong to a class of phospholipid double‐membrane closed vesicles, and these can be divided into exosomes, microvesicles, and apoptotic bodies according to their size and biological formation process.^[^ ^1^ ^]^ The International Society for Extracellular Vesicles meetings in 2018 and 2023 suggested that it would be more appropriate to use small extracellular vesicles (sEVs) rather than exosomes because exosomes are associated with biogenetic pathways and are difficult to identify among other EVs with similar size.^[^ ^2^ ^]^ Thus, we also used sEVs instead of exosomes here. sEVs are cup‐shaped EVs with a diameter of 30–200 nm.^[^ ^2a^ ^]^ sEVs can be secreted by almost all kinds of cells into body fluids (e.g., blood, urine, saliva, tears, and cerebrospinal fluid) and the interstitial space, and they carry multiple contents from donor cells, including proteins, nucleic acids, and lipids.^[^ ^3^ ^]^ sEVs released by donor cells can be taken up by recipient cells, and they subsequently release their contents to facilitate intercellular communication.^[^ ^4^ ^]^ The membrane structure of sEVs protects their contents from hydrolysis by enzymes, allowing them to serve as biomarkers for various diseases.^[^ ^5^ ^]^ In addition, sEVs possess low immunogenicity and are amenable to targeted modification, which endow them with significant potential for drug delivery applications.^[^ ^6^ ^]^ Overall, the investigation of sEVs and their contents holds considerable importance for elucidating the mechanisms of intercellular communication and for enhancing clinical diagnosis and therapy.

In the past few years, tissue‐derived sEVs (TDsEVs) have become a prominent focus in biomedical research because TDsEVs are considered to more closely represent in vivo cellular communication compared to sEVs isolated from cell culture supernatants and body fluids.^[^ ^7^ ^]^ This advantage is attributed to the fact that TDsEVs are obtained directly from tissues, offering a more accurate reflection of the complex cellular milieu and interactions within specific tissues. TDsEVs can also mediate long‐distance inter‐organ communication and enhance communication between neighboring cells.^[^ ^8^ ^]^ For instance, lung TDsEVs can promote the recruitment of bone marrow neutrophils during inflammation by transferring double stranded DNA,^[^ ^8b^ ^]^ and brown adipose TDsEVs are capable of delivering miRNAs that mediate cardiac protection.^[^ ^8c^ ^]^

However, the isolation of pure TDsEVs remains challenging mainly due to contamination by cellular vesicles from ruptured cells during TDsEV isolation and the lack of suitable separation methods for specific tissues to obtain high yields of pure sEVs. Therefore, the key requirements for isolating TDsEVs are to fully release TDsEVs from the interstitial space without causing cell rupture and to develop suitable separation methods for specific tissues.^[^ ^2b^ ^]^ There have been extensive studies seeking to develop isolation methods for TDsEVs from many tissues, including brain, metastatic melanoma, and adipose tissue.^[^ ^7^, ^8^, ^9^ ^]^ For releasing sEVs from tissues, previous studies utilized grinding or homogenization to dissociate tissue, but these lead to cell rupture and non‐sEV contamination.^[^ ^10^ ^]^ Enzymatic digestion, as a gentler method of tissue dissociation, is now widely recognized as an effective methods of releasing sEVs from tissues.^[^ ^7^, ^11^ ^]^ Collagenase and papain are the most commonly used tissue‐digesting enzymes, while enzyme types and concentrations vary in different tissues.^[^ ^9^, ^11^ ^]^ The reported separation methods for TDsEVs include ultracentrifugation (UC), density gradient ultracentrifugation (DGU), and size exclusion chromatography (SEC). The efficiency of sEVs obtained in different ways from the same tissue might be different,^[^ ^4^, ^9^ ^]^ and thus it is necessary to establish optimal TDsEV isolation methods for each specific tissue.

The cochlea, a snail‐shaped structure situated in the ventral part of the inner ear, is essential for mechanical signal transduction, nerve excitation transmission, and sound perception.^[^ ^12^ ^]^ It has been reported that sEVs secreted from utricular supporting cells (SCs) can protect hair cells (HCs) from neomycin damage, and pericyte‐secreted sEVs can promote the growth of spiral neuronal neurites and blood vessel branches by enhancing intercellular communication.^[^ ^13^ ^]^ These studies indicate that sEVs actively participate in intercellular communication in the inner ear. However, these studies are based on in vitro cell culture models, and the information obtained may be somewhat different from the in vivo situation. Thus far, there is a lack of research on cochlea‐derived sEVs (CDsEVs) needed for an in‐depth comprehension of the in vivo roles of inner ear sEVs in auditory function. Therefore, it is urgent to establish a feasible isolation method for CDsEVs in order to obtain valuable information about physiological characteristics of CDsEVs and regulatory roles and to explore the mechanisms involved in the auditory system.

In our previous study, we isolated CDsEVs from mice of different ages by grinding tissues followed by UC, and we found that the isolated sEVs were related to inner ear development and hearing function.^[^ ^14^ ^]^ However, considering that even the mildest grinding cannot completely avoid contamination by cellular contents, resulting in impurity of the extracted CDsEVs, hindering further functional research, optimal enzyme digestion methods should be established to avoid cell damage and release CDsEVs in tissue intercellular space to the greatest extent, and thus improve the purity of isolated CDsEVs for more accurate functional research. Additionally, we did not study the effect of different separation and isolation methods on the purity and yield of CDsEVs, which is shown previously to influence the purity and yield of sEVs in other tissues.^[^ ^9^, ^15^ ^]^ Therefore, in this study, we systematically studied the optimal methods for cochlear tissue digestion and CDsEV isolation from the cochlea of neonatal mice, and we further comprehensively analyzed the molecular profiles of the CDsEVs and their possible cellular origin.

## 2 Results

### 2.1 Comparing the Efficiency of Different Enzymes for Cochlear Tissue Dissociation

**Figure 1:**
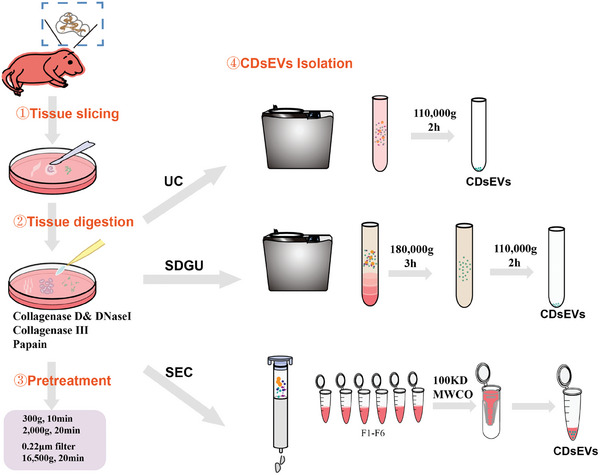
Flow chart of CDsEV isolation. ① The cochleae were dissected from the neonatal mice, separated into the basilar membrance (BM), modiolus (M), and spiral ligament (SL), and cut into small pieces. ② The pieces were digested with collagenase D combined with DNase I, with collagenase III alone, or with papain alone. Subsequently, an appropriate tissue digestion enzyme was selected. ③ The digested samples were pretreated to remove cell debris and large vesicles. ④ CDsEVs were isolated by UC, SDGU, or SEC.

In order to establish an efficient isolation method for CDsEVs, we systematically investigated various tissue dissociation enzymes and sEV isolation methods. First, we evaluated the digestion efficacy of collagenase D combined with DNase Ι, collagenase III alone, and papain alone in order to identify the optimal enzyme for digesting cochlear tissues. Following this, we used UC, sucrose density gradient ultracentrifugation (SDGU), and SEC to isolate CDsEVs, and we ultimately determined the most effective method for isolating and purifying CDsEVs through comparative analysis (**Figure** **1**).

**Figure 2:**
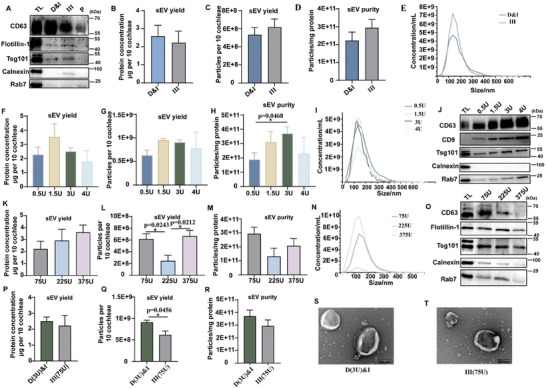
Comparison of the digestive efficiency of different enzymes on cochlear tissue for CDsEV isolation. A–E) The expression of the sEV marker proteins CD63, Flotillin‐1, and Tsg101 and the negative markers Calnexin and Rab7 were detected in CDsEVs by WB (A) after digestion with 0.5 U collagenase D combined with 40 U DNase Ι (D&I), 75 U collagenase III (III), or 20 U papain (P). Cochlear tissue total lysate (TL) was used as the positive control. The protein quantification (B), the particle numbers (C), the ratio of particle number to protein concentration (D), and the concentration and size distribution (E) of CDsEVs were detected and calculated. F–O) The protein quantification (F, K), the particle numbers (G, L), the ratio of particle number to protein concentration (H, M), the concentration and size distribution (I, N), and the EV marker protein analysis (J, O) of CDsEVs were detected and calculated after the digestion of cochlear tissue using different concentrations of collagenase D combined with 40 U DNase I (F‐J) or different concentrations of collagenase III (K‐O). P–T) The protein quantification (P), the particle numbers (Q), the ratio of particle number to protein concentration (R), and the transmission electron microscopy (TEM) morphology (S, T) of CDsEVs were compared between two enzymes. Scale bar, 200 nm. *p < 0.05. n = 3.

According to previously reported enzyme concentrations, we sliced postnatal day (P) 2–4 cochleae into ≈ 2 mm pieces and digested them separately using 0.5 U collagenase D combined with 40 U DNase Ι, 75 U collagenase III, or 20 U papain.^[^ ^12^ ^]^ The CDsEVs were isolated by the “gold standard” UC method. sEV‐positive protein markers CD63, Flotillin‐1, and Tsg101 were detected by western blotting (WB) in the 0.5 U collagenase D combined with 40 U DNase Ι and the 75 U collagenase III‐digested samples, but not in the 20 U papain‐digested samples (**Figure** **2**A), which suggested that papain is not suitable for cochlear tissue dissociation to isolate CDsEVs. We further compared the protein concentrations, particle numbers, and the ratios of particle numbers to protein concentration in 0.5 U collagenase D combined with 40 U DNase Ι‐digested samples and 75 U collagenase III‐digested samples using the bicinchoninic acid assay (BCA) kit and nanoparticle tracing analysis (NTA) (Figure 2B–D). The ratio of particle numbers to protein concentration serves as an indicator for assessing the purity of sEVs, and a higher ratio corresponds to a greater purity of sEVs.^[^ ^11^, ^16^ ^]^ These results showed no significant difference. The average particle size of CDsEVs in these two groups of samples was ≈ 150 nm, which is a typical sEV size (Figure 2E).

In order to determine the optimal digestive concentration of collagenase D and collagenase III for cochleae, we assessed the efficacy of different concentrations of these two enzymes. We first used 0.5 U, 1.5 U, 3 U, and 4 U collagenase D combined with 40 U DNase Ι to digest cochlear tissue and isolated the sEVs by UC. Total protein concentration and particle numbers of CDsEVs showed no significant difference, but the ratio of particle numbers to protein concentration was better in the 3 U group, indicating that the 3 U group yielded higher purity of CDsEVs (Figure 2F–H). The CDsEV particle size obtained in all groups was consistent with the typical sEV range of 30–200 nm (Figure 2I). The sEV protein markers CD63, CD9, and Tsg101 could be detected in all groups of CDsEVs by WB, and their intensity increased with the increase in enzyme concentration, while the negative marker Rab7 was highest in the 4 U sample (Figure 2J). Based on the above results, we ultimately chose 3U as the optimal concentration for collagenase D to digest cochlear tissue.

For collagenase III, we used three concentrations of 75 U, 225 U, and 375 U, and we also isolated sEVs by UC. Figure 2K–N showed that CDsEVs from the 75 U and 375 U collagenase III groups had a higher quantity of particles, with the 75 U group achieving the highest level of CDsEV purity. The WB results revealed that the intensity of sEV marker proteins was minimal in the 375 U group and highest in the 75 U group (Figure 2O). These results suggested that the 75 U collagenase III concentration was better for cochlear tissue digestion. Finally, we compared the digestion effects of 3 U collagenase D combined with 40 U DNase Ι and 75 U collagenase III. Although typical cup‐shaped sEVs could be obtained from both groups (Figure 2S,T), 3 U collagenase D combined with 40 U DNase Ι was slightly better in terms of protein concentration, particle number, and purity (Figure 2P–R). Together, these results suggested that both collagenase D and collagenase III can be used to digest cochlear tissue, and we finally chose 3 U collagenase D combined with 40 U DNase Ι as the relatively better choice for the subsequent experiments.

### 2.2 Comparing the Efficiency of Different Isolation Methods in CDsEVs

**Figure 3:**
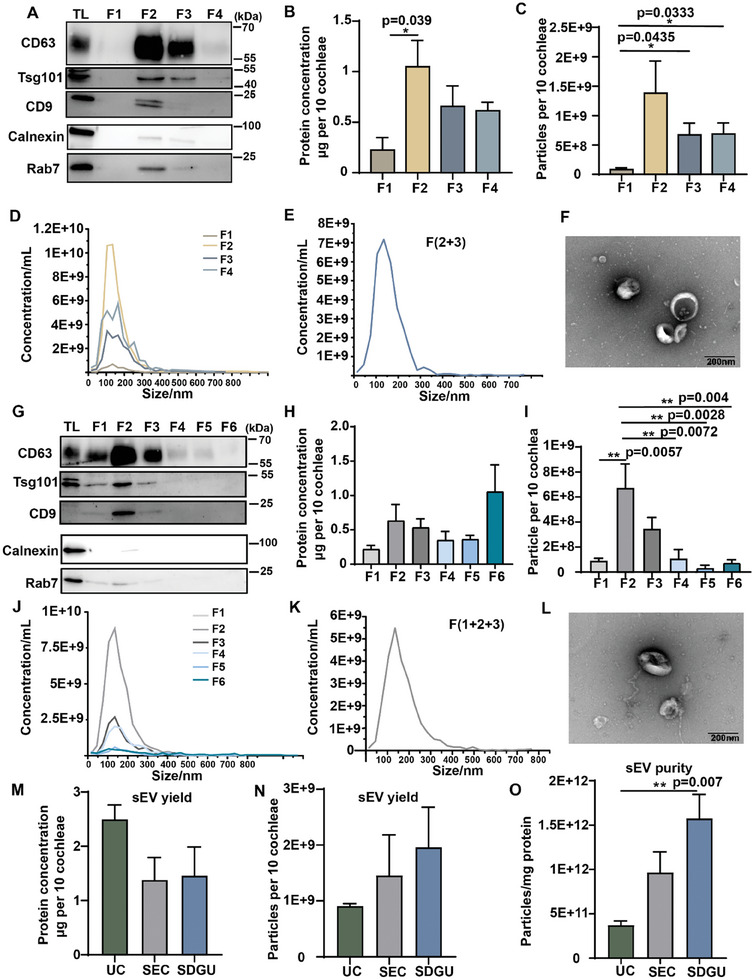
Comparison of the efficiency of different CDsEV isolation methods. A–D) The WB analysis of the sEV‐positive markers CD63, Tsg101, and CD9 and the negative markers Calnexin and Rab7 (A), the protein quantification (B), the particle numbers (C), and the concentration and size distribution (D) of F1–F4 obtained by SDGU. E,F) The concentration and size distribution (E) and TEM visualization (F) of the F2 and F3 mix obtained by SDGU. G–J) The WB analysis of sEV‐positive and negative markers proteins (G), the protein quantification (H), the particle numbers (I), and the concentration and size distribution (J) of F1–F6 obtained by SEC. K,L) The concentration and size distribution (K) and TEM visualization (L) of the F1, F2, and F3 mix obtained by SEC. M–O) The comparison of protein quantification (M), the particle numbers (N), and the ratio of particle number to protein concentration (O) of CDsEVs for the UC, SDGU, and SEC groups. Scale bar, 200 nm. *p < 0.05, **p < 0.01, n = 3.

Previous studies have demonstrated significant disparities in the yields of TDsEVs when isolated using various methods.^[^ ^9^, ^15^ ^]^ UC, SDGU, and SEC are commonly used sEV isolation methods, so we compared the yield of CDsEV isolated by these methods. For SDGU, cochlear samples pretreated with 3 U collagenase D combined with 40 U DNase Ι were laid onto a sucrose density gradient cushion with sucrose solutions of 0.6, 1.3, and 2.5 M  according to the density of sEVs.^[^ ^11b^ ^]^ Samples Fraction (F)1‐F4 were collected after UC at 180000 × *g*. **Figure** **3**A shows that the sEV marker proteins CD63, CD9, and Tsg101 were enriched in F2 and F3. Protein concentrations and particles were also higher in F2 and F3 (Figure 3B,C). Particles in F2 and F3 fractions showed typical sEV size ranging from 30 to 200 nm and typical cup‐shaped sEV morphology (Figure 3D; Figure S1, Supporting Information). The particle size and morphology of the F2 and F3 mixed sample were detected by NTA and TEM, and Figure 3E,F show that they had standard sEV size and morphological characteristics. These results indicated that CDsEVs were enriched in F2 and F3 by the SDGU isolation method, and we subsequently collected these two fractions as CDsEVs.

In addition, we also applied SEC to isolate CDsEVs. We added samples pretreated with 3 U collagenase D combined with 40 U DNase Ι to the SEC column, and six fractions were consecutively collected, namely F1‐F6. Figure 3G–J shows that CDsEVs were enriched in F1‐F3. CD63, CD9, and Tsg101 were enriched in F1‐F3 according to WB. The mean particle size of the F1‐F3 mixed sample was ≈ 150 nm, and the cup‐shaped structure could be observed by TEM (Figure 3K,L). These results suggested that CDsEVs were enriched in F1‐F3 by the SEC method, and we subsequently collected these three fractions as CDsEVs.

Finally, we compared the protein concentration and particle number of CDsEVs isolated by the UC, SDGU, and SEC methods. The protein concentration obtained by UC was the highest, while the number of particles obtained by SDGU was the highest (Figure 3M,N). The ratio of particle numbers to protein concentration was greatest in SDGU‐obtained CDsEVs, exceeding 2E+11 (Figure 3O), which suggested a higher purity of CDsEVs in accordance with previous studies.^[^ ^16^ ^]^ Based on all the above results, we propose that CDsEVs can be isolated most efficiently by SDGU after digesting the cochleae with 3 U collagenase D and 40 U DNase Ι.

### 2.3 Small‐RNA Seq Analysis of miRNAs in CDsEVs

**Figure 4:**
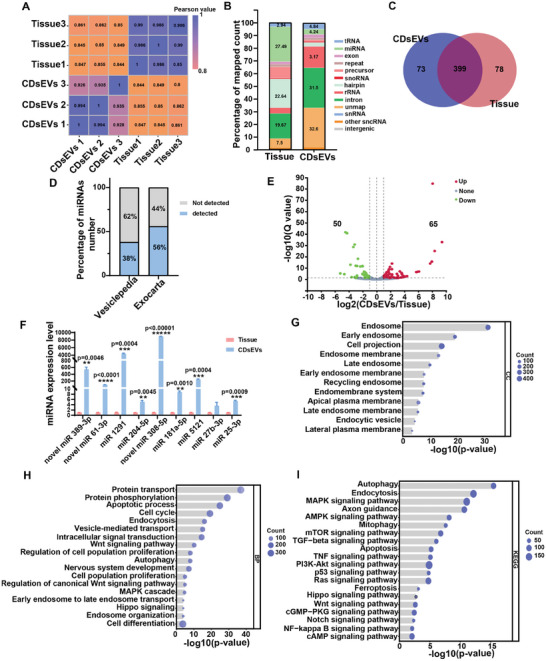
Small RNA sequencing analysis of CDsEVs. A,B) Cluster analysis (A) and small RNA type and percentage analysis (B) of CDsEV and cochlear tissue small RNA‐seq data. C) The Venn diagram of CDsEVs and cochlear tissue miRNA data. D) Analysis of the proportion of CDsEV miRNAs within the Vesiclepedia and Exocarta databases. E) The volcano map of differentially expressed miRNAs. FC >2, Q value < 0.05. F) Verification of differentially expressed miRNA by Q‐PCR. G–I) GO (G, H) and KEGG (I) analysis of target genes of enriched miRNAs in CDsEVs. The top cellular components (G) and biological processes (H) are shown. *p < 0.05, **p < 0.01, ***p < 0.001, ****p < 0.0001, *****p < 0.00001, n = 3.

sEV contents include nucleic acids, proteins, and lipids. Small RNAs, which play important roles in intercellular communications and disease diagnosis, are an important component of the nucleic acid fraction in sEVs.^[^ ^17^ ^]^ Therefore, we used small‐RNA seq to analyze small RNAs in CDsEVs, using the same amount of RNA from cochlear tissue as the control group. We calculated the pearson correlation coefficients between two samples, and the results showed that the samples were well distributed (**Figure** **4**A). The cochlear samples and CDsEV samples contained a variety of small RNAs, including tRNA, miRNA, snoRNA, rRNA, and other small RNA, with miRNAs accounting for 27.49% of all nucleic acids in the cochlear tissue group and 4.24% in the CDsEV group (Figure 4B). A total of 550 miRNAs were detected, of which 399 miRNAs were found in both groups, and 73 and 78 were found only in CDsEVs and cochlear tissue, respectively (Figure 4C). A total of 38% and 56% of miRNAs in CDsEVs were present in the Vesiclepedia and Exocarta databases, respectively, and the proportion of miRNAs was higher in the Exocarta database compared to the Vesiclepedia database (Figure 4D; Figure S2A, Supporting Information). We performed a significant differential expression analysis between the two groups using fold change (FC) > 2 and Q‐value < 0.05 as the criteria,^[^ ^13^, ^18^ ^]^ and we found that 65 miRNAs were increased and 50 miRNAs were decreased in CDsEVs compared to cochlear tissue (Figure 4E). We validated the top 9 increased miRNAs in CDsEVs by Q‐PCR, and the results were consistent with the sequencing results (Figure 4F).

To further explore the possible functions of miRNAs in CDsEVs, we performed Gene Ontology (GO) and Kyoto Encyclopedia of Genes and Genomes (KEGG) analyses of the predicted target genes of the 65 enriched miRNAs in CDsEVs. Cell component analysis suggested that the sEVs were mainly derived from endosomes, early endosomes, and late endosomes, which are closely related to the nuclear endosome system involved in sEV formation (Figure 4G). The GO biological process analysis suggested that these miRNAs are involved in nervous system development, vesicle‐mediated transport, and early endosome to late endosome transport, in important cellular functions such as cell proliferation, cell differentiation, cell cycle progression, and in signaling pathways like the Wnt, Hippo, and MAPK signaling pathways (Figure 4H). In addition, KEGG analysis found that the target genes of 65 CDsEVs‐derived miRNAs were involved in multiple auditory activity signaling pathways such as the AMPK, TGF‐β, Hippo, Notch, and MAPK signaling pathways (Figure 4I). These results indicated that our isolation method could effectively isolate sEVs from cochlear tissue, and thus miRNAs in CDsEVs might act as regulators in the process of auditory ontogeny and maturation.

### 2.4 Label‐Free Proteomic Profiling of CDsEVs

**Figure 5:**
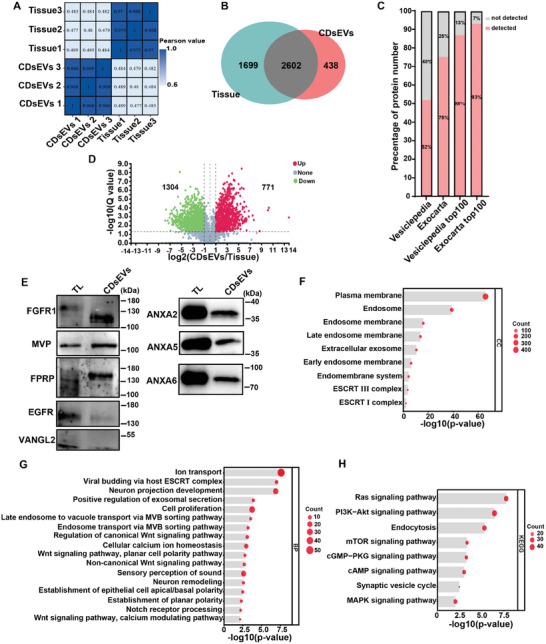
Proteomics analysis of CDsEVs. A,B) Cluster analysis (A) and Venn diagram (B) of the proteomics data for CDsEVs and cochlear tissue. C) Analysis of the proportion of proteins in CDsEVs within the Vesiclepedia and Exocarta databases. D) The volcano map of differentially expressed proteins. E) Verification of CDsEV‐enriched proteins by WB. F–H) GO (F, G) and KEGG (H) analysis of enriched proteins in CDsEVs. The top cellular components (F) and biological processes (G) are shown.

In order to explore the protein components and biological functions of CDsEVs, we conducted a label‐free quantitative proteomics analysis of CDsEVs, with the same amount of protein of cochlear tissues used as the control. Each group included three biological replicates with good correlation between samples (**Figure** **5**A). A total of 4739 proteins were identified in the two groups, with 3040 proteins identified in CDsEVs and 4301 proteins identified in cochlear tissues (Figure 5B). We also compared proteins in CDsEVs with the proteins in the Vesiclepedia and Exocarta databases and found that 52% of the proteins in CDsEVs could be found in Vesiclepedia and 75% of the proteins in CDsEVs could be found in Exocarta. Moreover, the top 100 proteins from Vesiclepedia and Exocarta accounted for 88% and 93% of the proteins in our data, respectively (Figure 5C; Figure S2B,C, Supporting Information). This high degree of overlap indicated that the CDsEVs isolated by our method possess a high level of purity. The volcano map showed the differentially expressed proteins in CDsEVs compared to cochlear tissue, with 771 proteins being significantly increased and 1304 proteins being significantly decreased (FC > 2, Q‐value < 0.05) (Figure 5D). We verified five highly enriched proteins in CDsEVs, including Fibroblast growth factor receptor 1 (FGFR1), major vault protein (MVP), prostaglandin F2 receptor negative regulator (FPRP), epidermal growth factor receptor (EGFR), and vang‐like protein 2 (VANGL2) (FC > 10, Q < 0.05), and three proteins with the highest expression level in CDsEVs, including annexin A2 (ANXA2), annexin A5 (ANXA5), and annexin A6 (ANXA6), by WB. FGFR1, EGFR, and VANGL2 are involved in the establishment of auditory function, and MVP and FPRP are involved in the loading of sEV contents.^[^ ^19^ ^]^ Figure 5E showed that five proteins – FGFR1, MVP, FPRP, ANXA6, ANXA2, and ANXA5 – were expressed in CDsEVs, among which FGFR1 and FPRP were enriched in CDsEVs compared to cochlear tissue.

We performed GO and KEGG analysis of 771 proteins that were enriched in CDsEVs to predict the biological functions of CDsEVs. These proteins were enriched in terms associated with EV‐like extracellular exosomes, endosomes, and ESCRT complexes according to GO cellular component analysis (Figure 5F), which also further demonstrated the high purity of our isolated CDsEVs. In terms of biological processes, we found that CDsEVs are involved in ion transport, neuron projection development, cell proliferation, Wnt signaling pathway, and the establishment of planar polarity, which are crucial for the early stages of hearing formation. Additionally, these proteins are also involved in the MVB sorting pathway and the regulation of exosome secretion, and more importantly they contribute to the perception of sound (Figure 5G). We also found that proteins in CDsEVs are involved in multiple signaling pathways like Ras, PI3K‐Akt, and cAMP and in the synaptic vesicle cycle (Figure 5H). These results indicated that proteins in CDsEVs actively participate in the biological processes of cochlear formation, the maturation of HCs, and the formation of synaptic connections during the development of hearing and have important significance for the establishment of the auditory system and sound perception.

### 2.5 Influence of FGFR1 in CDsEVs for HC Survival

**Figure 6:**
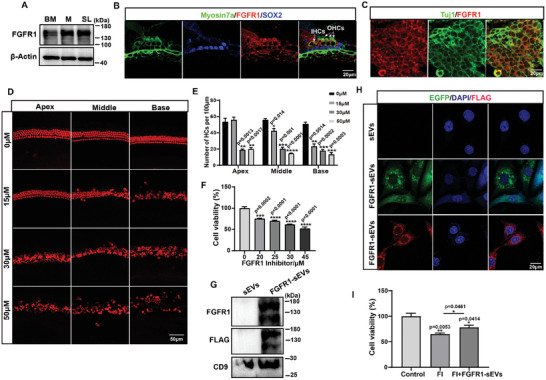
Expression and roles of CDsEV FGFR1. A–C) The expression of FGFR1 in the BM, M, and SL as measured by WB (A) and immunofluorescence staining in the OC (B) and SGN (C) in cochleae from P3 mice. Myosin7a (green) and SOX2 (blue) were used as HC and SC markers, respectively, in (B), and Tuj (green) was used as the SGN marker (C). Scale bar, 20 µm. D,E) Immunofluorescence staining (D) of BM explants treated with different concentrations (0, 15, 30, 50 µm) of the FGFR1 inhibitor PD166866 in vitro. Myosin7a (red) was used as the HC marker. Scale bar, 50 µm. Quantification of HC numbers per 100 µm of the apical, middle, and basal turns of BM explants (E). F) HEI‐OC1 cell viability with different concentrations of FGFR1 inhibitor PD166866 treatment. G) WB analysis of FGFR1‐overexpressing sEVs (FGFR1‐sEVs) obtained from the culture medium of 293T cells transfected with FGFR1‐EGFP‐FLAG plasmids. H) Immunofluorescence staining of EGFP and FLAG in HEI‐OC1 cells incubated with FGFR1‐sEVs. Scale bar, 20 µm. I) Cell viability of HEI‐OC1 cells treated with FGFR1 inhibitor (FI) and FGFR1‐sEVs. *p < 0.05, **p < 0.01, ***p < 0.001, ****p < 0.0001, n = 3.

FGFR1 is the surface receptor protein for fibroblast growth factor, which participates in the perception of sound and in the morphogenesis and development of auditory cells in the inner ear.^[^ ^19^, ^20^ ^]^ FGFR1 was significantly enriched in CDsEVs as our data showed above, suggesting that FGFR1 in CDsEVs might be involved in the normal function of the inner ear. We first detected FGFR1 expression in the basilar membrane (BM), modiolus (M), and spiral ligament (SL) of the cochlea by WB and found that FGFR1 was widely expressed in the inner ear (**Figure** **6**A). Subsequently, immunofluorescence staining also confirmed this result (Figure S3A, Supporting Information), which showed that FGFR1 was mainly localized in HCs rather than SCs in the organ of Corti and was expressed in spiral ganglion neurons (SGNs) (Figure 6B,C). Supporting Information Figure S3B,C (Supporting Information) show that FGFR1 was expressed in the cytoplasm and kinocilium roots of HCs.

To further investigate whether FGFR1 affects cellular function in the cochlea as previously reported, different concentrations of the FGFR1 inhibitor PD‐166866 were added into culture medium of BM explant in vitro, and the results showed that HCs were significantly damaged from the basal turn to the middle turn even with the lowest concentration of FGFR1 inhibitor treatment (15 µM PD‐166866). The loss of nerve fibers was also observed along with the damage of HCs, and their damage and loss gradually increase as the inhibitor concentration increase (Figure 6D,E; Figure S3D,E, Supporting Information). We also treated HEI‐OC1 cells with different concentrations of the FGFR1 inhibitor PD‐166866 and found that the cell viability of HEI‐OC1 cells was decreased after treatment with the inhibitor in a dose‐dependent manner (Figure 6F). Subsequently, we constructed an FGFR1‐EGFP‐FLAG overexpressing plasmid and overexpressed FGFR1 in HEK‐293T cells (Figure S4A,B, Supporting Information). Figure 6G shows that FGFR1 was successfully overexpressed in sEVs isolated from the FGFR1‐overexpressing HEK‐293T cell culture medium. Supporting Information Figure S4C,D (Supporting Information) show the particle size distribution of sEVs. EGFP fluorescence and FLAG tag could be observed when these FGFR1‐overexpressing sEVs (FGFR1‐sEVs) were added to the HEI‐OC1 cells (Figure 6H). Importantly, we found that the degree of reduction in HEI‐OC1 viability caused by FGFR1 inhibitor treatment could be alleviated by adding FGFR1‐sEVs (Figure 6I). These results suggested that impaired FGFR1 expression affects HC survival and that cell damage caused by FGFR1 inhibition can be alleviated by FGFR1 via sEVs.

### 2.6 Combined Analysis of Single sEV‐seq Data and Cochlear scRNA‐seq Data to Trace the Cellular Origin of CDsEVs

**Figure 7:**
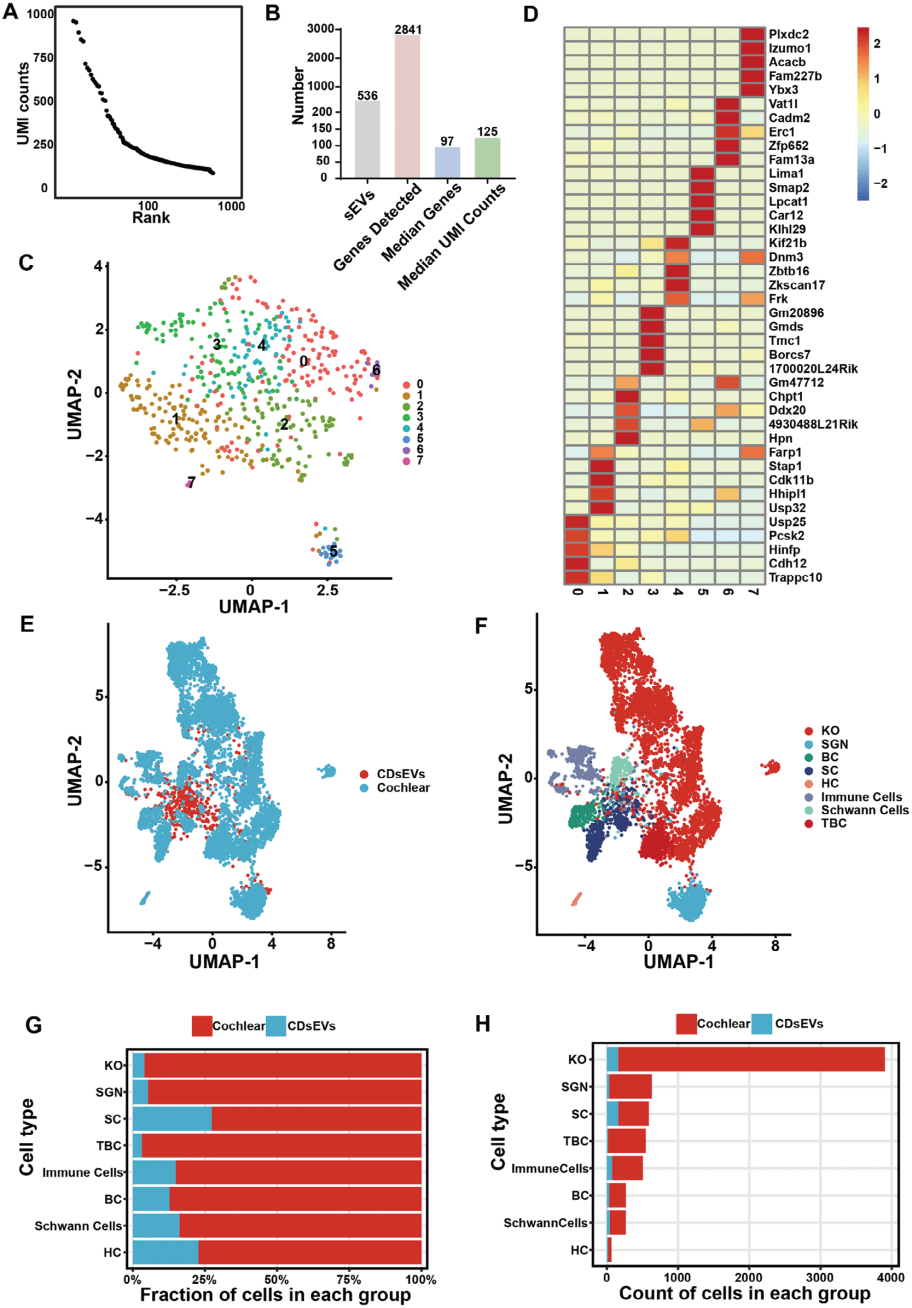
Single CDsEV transcriptomes and combined analysis with cochlear scRNA‐seq. A) Barcode rank plots showing the distribution of UMI counts associated with each barcode in the filtered matrix. B) The number of qualified CDsEVs, total detected genes, median number of genes, and median UMI counts in each CDsEV. C) UMAP visualization of the cluster profile for the single CDsEV‐seq data. D) The heat map showing the five genes that were highly expressed in each CDsEV cluster. E,F) UMAP visualization of the CDsEVs and cochlear cells (E) and cluster profile (F) for the integrated dataset of single CDsEV‐seq and cochlear scRNA‐seq. G,H) The bar chart shows the proportions (G) and count numbers (H) of the CDsEVs and cochlear cells in each cluster of the integrated dataset of single CDsEV‐seq and cochlear scRNA‐seq. Kölliker's organ cells, KO, Spiral ganglion neuron cells, SGN, Basal cells, BC, Supporting cells, SC, Hair cells, HC, Tympanic border cells, TBC.

A recent study showed that sEVs can be sequenced individually and grouped into sEV clusters, just like single‐cell RNA sequencing (scRNA‐seq) data.^[^ ^21^ ^]^ Considering that analyzing the bulk data of sEVs can result in the loss of individual sEV heterogeneity, we performed single CDsEV sequencing to further investigate the heterogeneity of single CDsEVs and then traced their cell origin by combined analysis with cochlear scRNA‐seq data. Referencing Luo et al.’s previous research on single EV sequencing and analysis,^[^ ^21a^ ^]^ we first performed a single EV sequencing analysis of CDsEVs using the 10× Genomics sequencing platform. Due to the smaller size of CDsEVs compared to cells, the cell‐calling algorithm in Cell Ranger in the 10× Genomics platform failed to reveal distinct inflection points (Figure S5A, Supporting Information). However, these inflection points became apparent after processing with the CB2 algorithm (**Figure** **7**A). After that, a total of 536 qualified sEVs were detected, and 2841 genes were found to be expressed in the CDsEVs. The median number of genes per sEV was 97, and the median unique molecular identifier (UMI) count was 125 (Figure 7B). The frequency distribution of gene numbers and UMI counts in CDsEVs are shown in Figure S5B,C (Supporting Information). To investigate the transcriptional heterogeneity within individual EVs, we analyzed single sEV data and identified eight clusters (Figure 7C), each of which contained a different number of sEVs (Figure S5D, Supporting Information) and might represent different cell origins, activation states, or biological processes. Figure 7D shows the top five genes of each sEV cluster.

To trace the cell origin of the CDsEVs, we further integrated the single CDsEV‐seq data with the scRNA‐seq data of the cochlea (GSE135913) and identified eight clusters, each of which contained both sEVs and cells, suggesting that the CDsEVs originated from many different cell types (Figure 7E,F). Supporting Information Figure S5E (Supporting Information) shows the five marker genes that were highly expressed in each cluster. Among the eight clusters, the proportion of sEVs in the SC cluster was the highest (38%, Figure 7G), which indicated that the number of sEVs secreted per cell was the highest in SCs. In terms of total numbers of sEVs in each cluster, SCs and Kölliker's organ (KO) cells contained the highest number of sEVs, which suggests that SCs and KOs secreted more sEVs than other clusters (161 and 158 sEVs in SCs and KOs, respectively, Figure 7H). These results suggested that SCs and KOs have the most robust capacity for CDsEV secretion and are the main source of CDsEVs.

## 3 Discussion

In this study, we established an optimal method for isolating neonatal CDsEVs, and we identified the contents and analyzed the cell origins of CDsEVs. Compared with our previous study,^[^ ^14^ ^]^ we optimized the release of sEVs from cochlear tissues using enzymatic digestion, which reduces cellular contaminants produced by tissue grinding. Moreover, we observed that different isolation methods in the cochlea influence both the purity and yield of EVs, especially that the purity of CDsEVs isolated by SDGU method is significantly higher than by UC method and is the only one that exceeds 2E+11. These all suggests that the enzyme digestion and SDGU method improves the isolation purity and yield compared with previous work, which will facilitate further functional research of CDsEVs. Moreover, our findings suggest that the miRNAs and proteins of CDsEVs may be involved in the maintenance of auditory function, and we found that cochlear cells exhibited variable capacities for secreting sEVs.

As an important intercellular communication medium, sEVs are involved in many physiological and pathological processes.^[^ ^22^ ^]^ Many studies have suggested that sEVs derived from tissues are more effective at reflecting intercellular communication mediated by sEVs in specific tissues compared to sEVs isolated from cell supernatants and body fluids.^[^ ^8^, ^9^ ^]^ Previous studies have demonstrated that sEVs can be isolated from cochlear explants and in vitro cell culture supernatants.^[^ ^13^ ^]^ In this study, we performed a systematic quantitative and qualitative comparison of CDsEVs obtained by commonly used tissue dissociation enzymes and sEV isolation methods. The first enzyme used for TDsEVs isolation was papain, but papain lacks substrate specificity, which may lead to the digestion of EV surface proteins and affect downstream analysis.^[^ ^9^, ^10^ ^]^ Subsequently, collagenase III and D have been introduced as alternative enzymes for TDsEVs extraction.^[^ ^7^, ^9^, ^15^ ^]^ These collagenases selectively cleave components of the extracellular matrix, thereby preserving sEVs integrity by avoiding nonspecific degradation.^[^ ^11a^ ^]^ We used papain, collagenase D, and collagenase III to digest the tissues and found that papain was not suitable for cochlear tissue digestion to isolate CDsEVs, while there was no significant difference in the digestion effects of the other two collagenases. Therefore, either collagenase D or collagenase III can be used for cochlear digestion for CDsEV research. We ultimately selected 3 U collagen D due to its slightly better digestive efficacy compared to that of collagenase III at its optimal concentration. Studies have shown different efficiencies of sEV isolation by using different isolation methods.^[^ ^23^ ^]^ Zhang et al. found that higher purity of human brain‐derived sEVs is isolated by SDGU.^[^ ^15^ ^]^ Huang et al. showed that using SEC alone cannot achieve the purity of SDGU, but its combination with ultrafiltration (UF) can achieve higher efficiency than SDGU.^[^ ^9b^ ^]^ SDGU is more time‐consuming although the purity of isolated sEVs is higher, while SEC preserves the structure and integrity of sEVs better, but is not suitable for high‐throughput sample extraction. UC is also widely used for the separation of TDsEVs such as kidney and lung, but non‐sEV particles of similar size may be co‐isolated.^[^ ^8^, ^11^ ^]^ We further compared the methods of UC, SDGU, and SEC for isolating CDsEVs through characterization and quantitative analysis of CDsEVs, and found that SDGU exhibited superior performance in terms of both particle numbers and sEV purity. Based on these results, we proposed that the use of 3 U collagenase D combined with 40 U DNase Ι in conjunction with SDGU can effectively isolate CDsEVs, and this protocol will be helpful for subsequent CDsEVs research.

Many studies have indicated that the miRNAs and proteins of sEVs are involved in numerous biological processes, including intercellular communication, immune responses, and metabolic pathways.^[^ ^22^, ^24^ ^]^ To investigate the function of miRNAs and proteins in the CDsEVs, we conducted small RNA‐seq and proteomics analysis. We identified a total of 472 miRNAs in CDsEVs, and we validated 9 up‐regulated miRNAs in CDsEVs by Q‐PCR. MiR 204–5p, miR 181a‐5p, miR 27b‐3p, and miR 25‐3p participate in cell proliferation, differentiation, and migration via sEVs.^[^ ^25^ ^]^ MiR 1291 is a pivotal factor in the regulation of cancer cells, significantly affecting their proliferation, invasion, and apoptosis.^[^ ^26^ ^]^ Levels of miR 5121 in exosomes derived from microglia have been shown to influence synaptic growth and recovery, and overexpression of miR 5121 can promote neurite growth and synaptic recovery.^[^ ^27^ ^]^ However, miR 389‐3p, miR 61–3p, and miR 308‐5p are novel miRNAs and have no reported function, which needs further study. The process of auditory transmission requires proper synaptic connections. Whether miR 5121 in CDsEVs is related to synaptic growth and whether miR 5121 can be regulated to restore hearing impairment caused by aberrant synaptic connections is worthy of subsequent research. Additionally, these validated miRNAs associated with cell proliferation and differentiation may be potential candidates for regulating HC regeneration and function.

Protein profiling revealed the presence of 3040 proteins in CDsEVs, with 771 proteins being significantly enriched in CDsEVs. We compared the protein profile data of CDsEVs with the EV databases and found that over half of the data was present in the Exocarta database. Notably, 93% of the top 100 proteins listed in Exocarta were identified in the CDsEVs. These findings suggest that our proposed method effectively isolated CDsEVs. Furthermore, our functional prediction analysis of the 771 enriched proteins revealed their roles in contributing to the maturation of HCs, the innervation of neurons, synaptic vesicle transit, and sound perception. We further validated eight proteins by WB, and FGFR1, MVP, FPRP, ANXA2, ANXA5, and ANXA6 were found to be expressed in CDsEVs, with FPRP and FGFR1 being notably enriched in CDsEVs, while EGFR and VANGL2 were not expressed in CDsEVs.

FGFR1 is a surface receptor protein that belongs to the family of fibroblast growth factors. As a member of the tyrosine kinase receptor family, FGFR1 assumes a pivotal role in a multitude of biological processes, including development, cell proliferation, differentiation, and migration.^[^ ^28^ ^]^ Additionally, it contributes to sound perception, inner ear morphogenesis, and auditory cell development.^[^ ^19^, ^29^ ^]^ In mice lacking FGFR1, there is a reduction in the number of HCs and SCs in the cochlea, and the development of the entire cochlea is abnormal during embryonic stages.^[^ ^19^, ^29^ ^]^ FGFR1 also influences cilia polarity in the cochleae of chickens.^[^ ^20b^ ^]^ Our results indicated that FGFR1 is broadly expressed throughout the cochlea and is predominantly localized to HCs within the OC. Additionally, we observed that the inhibition of FGFR1 in vitro led to damage of HCs in cochlear explants and a reduction in HEI‐OC1 cell viability. Our findings further demonstrated the critical role of FGFR1 in maintaining HC survival. Interestingly, the addition of FGFR1‐sEVs could alleviate the phenomenon of reduced HEI‐OC1 cell viability caused by FGFR1 inhibitor. Studies have shown that FGF2 can activate the signal transduction of FGFR1β, leading to the activation of PI3‐K/Akt, thereby inhibiting the apoptosis of leukemic cells and promoting cell survival.^[^ ^30^ ^]^ Subsequently, Nathalie et al. found that autocrine FGF2‐FGFR1 activation in bone marrow stromal cells increased the secretion of exosomes containing FGF2, which supported leukemic cell survival. This protective effect was diminished when FGFR1 was inhibited, but it is still unclear how FGFR1 and FGF‐2 bind in exosomes.^[^ ^31^ ^]^ In the microenvironment of esophageal cancer cells, FGF‐2 mediates FGFR1 signaling to regulate the survival of macrophages and cancer cells.^[^ ^32^ ^]^ Furthermore, FGFR1 can also stimulate the AKT and MAPK pro‐survival signaling pathways to ensure cell survival.^[^ ^33^ ^]^ We speculate that the enrichment of FGFR1 in CDsEVs may protect HCs by releasing FGFR1 to activate the PI3‐K/Akt and MAPK pro‐survival signaling pathways, particularly when HC damage is induced by FGFR1 deficiency, but all of this needs further investigation.

Considering that the cochlea is a complex structure composed of multiple cell types, the CDsEVs we isolated are still a mixture of sEVs secreted from several cell types. Therefore, we performed single sEV‐seq for CDsEVs and conducted a joint analysis with the scRNA‐seq data of the cochlea to try to determine the cell origin of these CDsEVs. Our data showed that high‐quality CDsEVs can be obtained, sequenced, and clustered, just as is done for scRNA‐seq and analysis. Moreover, single sEV‐seq data can be integrated with cochlear scRNA‐seq data and clustered into different cell types. Each cluster contains cells and sEVs, which can be regarded as sEVs and their original cells. Our results showed that SCs had the highest sEV‐secretion efficiency, while SCs and KOs were the clusters secreting the greatest number of sEVs. We also conducted a joint analysis of CDsEV MS data and cochlear scRNA‐seq data (Figures S6–S8, Supporting Information) as previously reported,^[^ ^8b^ ^]^ and we also found that the KO cell cluster secreted many more sEVs than the other clusters (Figure S8F, Supporting Information), which is consistent with the single CDsEV‐seq analysis. However, in terms of the average number of sEVs each cell secreted, SGN cells exhibited superior capacity for CDsEV secretion in the joint MS data analysis (Figure S8D, Supporting Information), which might be because we currently can obtain only a relatively small number of sEVs resulting in a larger error in the estimation of sEV secretion from each cell. Considering that the expression of proteins and mRNAs of the same gene are not always consistent and that the MS data is the bulk expression data of sEV proteins, we believe that single sEV‐seq data is more reliable and accurate because it provides expression information at the single sEV level. Upon conducting an integrated analysis of single CDsEV data with scRNA‐seq data, we observed that individual HCs have a secondary capacity for CDsEV secretion that is weaker than that of SCs (Figure 7G). It is well known that HCs in the cochlea are an important type of sensory cell, and SCs have the ability to redifferentiate and can act as progenitor cells for HCs.^[^ ^34^ ^]^ This unique functional characteristic may underlie their higher sEV secretion to facilitate cellular communication within the cochlea and thus maintain normal auditory function, which also indicates the direction for our future research.

Overall, in this study we propose an optimal method for the isolation of CDsEVs and show that CDsEVs are closely involved in the auditory function of the cochlea. The optimal method for isolation of CDsEVs provided technical details for other researchers to further study the intercellular communication of CDsEVs and to reveal the possible roles of mechanisms of CDsEVs in the cochlea. The miRNAs and proteins we found enriched in CDsEVs, especially those miRNAs and proteins that we confirmed their expression in CDsEVs by QPCR and western blotting, provide targets and hints for the future further study of CDsEVs to elucidate their specific roles. Moreover, FGFR1 protein we found enriched in CDsEVs, which may be involved in HC survival, provides a new study target of HC protection and may help discover new therapeutic treatment for hearing loss. The cell origin study results of CDsEVs provide hints for further study of CDsEVs from different cell types of cochleae and to further explore the intercellular communication in the cochlea in which sEVs participate. All these preliminary observations could provide new insights into hearing function and its disorders.

## 4 Experimental Section

### Cochlear Tissue Enzyme Digestion and CDsEV Isolation

Cochlear tissue was obtained from ≈ 50 wild‐type neonatal mice (P2‐4). The cochlear shells were removed, and the cochleae were dissected and separated into the BM, M, and SL and then cut into ≈ 2 mm pieces in cold Dulbecco's Modified Eagle Medium (DMEM, Gibco, 11995500). Tissue slices were digested with 75 U, 225 U, or 375 U collagenase III (Worthington, LS004180) at 37 °C for 30 min, 0.5 U, 1.5 U, 3 U, or 4 U collagenase D (Roche, 11088858001) combined with 40 U DNase Ι (Sigma, 11284932001) at 37 °C for 45 min, or 20 U papain (Worthington, LS003119) at 37 °C for 30 min based on protocols from previous TDsEV studies.^[^ ^10^, ^11^ ^]^ During digestion process, the samples were placed on a constant temperature shaker with a rotating speed of 200 rpm. The samples were placed on ice immediately after digestion. cOmplete Protease Inhibitor (Roche, 04693132001) and PhosStop (Roche, 4906845001) solutions were added to stop collagenase III digestion, and cOmplete Protease Inhibitor was added to stop papain digestion. Digested tissue was filtered by a cell sieve with 40 µm pore size. Then the samples were centrifuged at 300 × *g* for 10 min, centrifuged at 2000 × *g* for 20 min at 4 °C, and filtered through a 0.22 µm filter to remove cell debris. The supernatant was spun at 16500 × *g* (referred to as the 16.5K supernatant) for 20 min at 4 °C to remove large vesicles. The precipitate was removed and the supernatant was subjected to UC, SDGU, and SEC for isolation of CDsEVs.

For UC, the 16.5K supernatant was centrifuged at 110000 × *g* for 2 h at 4 °C. Then the supernatant was removed and CDsEV pellets were resuspended with 150 µL DPBS (Solarbio, D1040) and stored at −80 °C.

For SDGU, sucrose solutions of 2.5, 1.3, and 0.6 M were prepared and gently added in order into the ultracentrifuge tube to form a sucrose cushion. The 16.5 K supernatant was then gently added to the sucrose cushion and DPBS was gently added to achieve a volume of 7.5 mL. Then the samples were centrifuged at 180000 × *g* for 3 h at 4 °C. After removing 6.3 mL supernatant, 1.2, 1.2, 1.2, and 1 mL sample fractions were collected as F1, F2, F3, and F4, separately. These four fractions were diluted with DPBS and centrifuged at 110000 × *g* for 70 min at 4 °C. Finally, the CDsEV pellets were resuspended with 150 µL DPBS.

For SEC, the 16.5 K supernatant was concentrated to 500 µL using a 100 KD ultrafiltration tube (Millipore, UFC810024). The 500 µL sample was added to the SEC column (qEVoriginal‐35 nm, IZON science), which was pre‐rinsed with 20 mL DPBS. When the sample entered the sieve plate of the column, and the liquid in the separation column stop dripping, DPBS continued to be added. After discarding the initial 3 mL of liquid drippings, every 500 µL droplet was collected consecutively as F1–F6. Finally, the six fractions were concentrated separately to ≈ 150 µL using a 100 KD MWCO ultrafiltration tube.

### Western Blotting (WB)

sEVs were lysed in 150 µL strong RIPA buffer (Beyotime, P0013B) supplemented with cOmplete Protease Inhibitor (Roche, 4693116001) for 30 min on ice, and 40 µL samples were taken for protein quantification using a BCA kit (Beyotime, P0010). The remaining samples were mixed with SDS loading buffer (Epizyme, LT101) and boiled for 15 min at 95 °C. The same amounts of total protein ≈ 4–10 µg from cochlear tissues and CDsEVs were separated by 4–15% SDS‐PAGE gradient gels (Beyotime, P0465S) and transferred onto PVDF membranes (Millipore, ISEQ00010) at 275 mA for 90 min. After blocking in 5% non‐fat milk solution (w/v) for 1 h at room temperature (RT), the membranes were incubated overnight with anti‐CD63 (Abcam, ab217345, 1:2000 dilution), anti‐CD9 (Abcam, ab92726, 1:2000, Santa Cruz, sc13118, 1:1000 dilution), anti‐Tsg101 (Abcam, ab125011, 1:2000 dilution), anti‐Flotillin‐1 (Cell Signaling Technology, 3253S, 1:1000 dilution), anti‐Calnexin (Abcam, ab22595,1:2000 dilution), anti‐Rab7 (Cell Signaling Technology, 9367, 1:1000 dilution), anti‐FGFR1 (Cell Signaling Technology, 9740S, 1:100 dilution), anti‐MVP (Abclonal, A1039, 1:500 dilution), anti‐FPRP (R&D systems, MAB10043‐SP, 0.1µg mL^−1^ dilution), anti‐EGFR (Abcam, ab52894, 1:1000 dilution), anti‐VANGL2 (Proteintech, 21492‐1‐AP, 1:1000 dilution), anti‐ANXA2 (Proteintech, 11256‐1‐AP, 1:1000 dilution), anti‐ANXA5 (Proteintech, 11060‐1‐AP, 1:2000 dilution), anti‐ANXA6 (Proteintech, 12542‐1‐AP, 1:1000 dilution), and anti‐FLAG (Sigma, F3165, 1:3000 dilution) primary antibodies. The next day the membranes were washed three times for 10 min in Tris‐buffered saline with Tween‐20 (TBST) and then incubated with HRP‐conjugated secondary antibody (Abmart, M21001 or M21002, 1:2000 dilution) at RT for 2 h. After washing three times for 10 min in TBST, SuperSignal West Pico Plus chemiluminescent substrate (Thermo Fisher, 34580) was added to detect the target bands. The images were captured on a Tanon‐5200 automated imaging system.

### Immunofluorescent Staining

The cochlear tissues and cells were fixed using a 4% paraformaldehyde solution for 1 h at RT. After washing with PBST [1× PBS with 0.1% Triton X‐100 (Solarbio, 1109F0521)] three times, the samples were mixed with blocking solution [PBS with 10% donkey serum (Solarbio, SL050), 1% Triton X‐100, and 1% BSA (Biofroxx, 4240)] and incubated at RT for 1 h. The samples were then incubated with the primary antibodies overnight. The following day, 4′,6‐diamidino‐2‐phenylindole (DAPI) (Solarbio, C0060) and fluorescent secondary antibodies that bind to the primary antibody were added. The fluorescent images of the cochleae and cells were captured with a Zeiss LSM 700 and 900 confocal microscopes. The primary antibodies used in this study were anti‐Myosin7a (Proteus Bioscience, 25–6790, 1:1000 dilution, DSHB, 138‐1, 1:400 dilution), anti‐Sox2 (R&D Systems, AF2018‐SP, 1:1000 dilution), anti‐FGFR1 (Cell Signaling Technology, 9740S, 1:100 dilution), anti‐Tuj1 (Beyotime, AT890, 1:400 dilution), anti‐acetylated tubulin (Proteintech, 66200‐1‐Ig, 1:100 dilution), and anti‐FLAG (Abclonal, AE092, 1:50 dilution). The fluorescent secondary antibodies included Alexa Fluor 647 donkey anti‐goat IgG (Invitrogen, A‐21447, 1:400 dilution), Alexa Fluor 555 donkey anti‐rabbit IgG (Invitrogen, A‐31572, 1:400 dilution), and Alexa Fluor 488 donkey anti‐mouse IgG (Invitrogen, A‐21202, 1:400 dilution).

### Nanoparticle Tracing Analysis (NTA)

sEV particle size distribution and concentration were measured by Zetaview Particle Matrix in scatter mode (488 nm laser) and with the shutter set to 70 using the Zetaview version 8.05.11 software (Zetaview Twin, Germany). After calibration with standard solution, sEVs were diluted to 1 mL with PBS (Procell, PB180327) and injected into the Zetaview instrument for detection. The measurement mode was set with a size distribution of 2 cycles, and the analysis parameters were set as max area 1000, min area 5, and min brightness 20. The Origin 8.5 software was employed for the analysis and processing of the data.

### Transmission Electron Microscopy (TEM)

sEVs were added to a copper grid for 10 min at RT. The sEVs were negatively stained with 3% (v/v) uranyl acetate solution (Solarbio, G1872) for 1–2 min and then dried at RT after removing the excess staining solution. The morphology of the sEVs was visualized by TEM (JEM‐2100).

### RNA Extraction and Real‐Time Q‐PCR

The sEVs’ miRNAs were extracted using a miRNA kit (Qiagen, 217184) according to the manufacturer's instructions. Briefly, sEVs were first dissolved in QIAzol lysis reagent. After the addition of chloroform, the lysate was centrifuged and divided into aqueous and organic phases. RNA was in the upper aqueous phase, DNA was in the mesophase, and proteins were in the lower organic phase or mesophase. The upper water phase was removed and mixed with ethanol, and the sample was added to the centrifuge column and the RNA was bound to the membrane. The RNA was eluted with RNase‐free water. Subsequently, the total RNA was reverse‐transcribed to obtained cDNA using a cDNA synthesis kit (Vazyme, MR‐101). Real‐time Q‐PCR was conducted on an Applied Biosystems real‐time PCR instrument using SYBR Master Mix (Vazyme, MQ‐101). The primers were designed using miRNA Design V1.01 and are listed in Supporting Information Tables S1 and S2 (Supporting Information). The data were normalized to U6.

### Small RNA Sequencing and Data Analysis

Small RNA sequencing was conducted by BGI Tech Solutions Co., Ltd, Guangdong, China, and the main process was as follows. Small RNA libraries were constructed by using 50 ng RNA from both CDsEVs and cochlear tissue. The RNA was combined with 3′ and 5′ adapters. Then the RNA was prepared for reverse transcription and PCR reaction, and the PCR product was denatured to a single strand. A cyclization reaction system was developed to obtain single‐stranded cyclized products. Single‐stranded circular DNA molecules underwent replication via rolling cycle amplification, resulting in the formation of a DNA nanosphere that encompassed multiple copies. The DNA nanospheres were loaded into patterned nanoarrays using the high‐intensity DNA nanochip technique, followed by sequencing by combinatorial Probe‐Anchor Synthesis.

Clean data were mapped to the reference genome and other small RNA databases including miRbase and the siRNA, piRNA, and snoRNA databases in Bowtie to identify miRNAs and other small RNAs.^[^ ^35^ ^]^ miRNA data were further analyzed in the Dr. Tom system from BGI, including principle component analysis (PCA) and differential expression analysis (FC > 2, Q‐value < 0.05). All miRNAs from CDsEVs were compared with the Vesiclepedia and Exocarta databases. The miRNAs’ target genes were predicted with the Tarbase v7.0 database. GO analysis was used to describe the cellular components and biological processes, and the KEGG database was used to annotate protein enrichment signaling pathways.^[^ ^36^ ^]^ Small RNA sequencing raw data have uploaded to NCBI SRA database (BioProject: PRJNA1168682).

### Mass Spectrometry (MS) and Proteomics Analysis

Label‐free quantitative proteomics analysis was conducted by BGI Tech Solutions Co., Ltd, Guangdong, China, and the main process was as follows. For the MS, 12 µg protein from CDsEVs and cochlear tissue was used as the starting material. The samples were digested into peptides with trypsin, and peptides were frozen and dried. The freeze‐dried peptides were redissolved with mobile phase A (2% acetonitrile, 0.1% formic acid) and centrifuged at 20000 × *g* for 10 min, and the supernatant was subjected to UHPLC (UltiMate 3000, Thermo). The samples were separated in series by a self‐loaded C18 column (75 µm inner diameter, 3 µm column particle size, 25 µm column length) at a flow rate of 300 nL mi^−1^n across a mobile phase gradient after being initially enriched and desalted in a trap column. The liquid phase‐separated peptide segments were ionized using a nanoESI source and entered the series mass spectrometer Q‐Exactive HF X (Thermo Fisher Scientific). MaxQuant was used to identify the raw data. Then MaxQuant conducted quantitative analysis based on peak intensity, peak area, and retention time of peptide segment related to primary MS, and conducted a series of statistical analysis and quality control.

The proteins identified by MaxQuant were further analyzed using the Dr. Tom system for PCA and differential expression analysis (FC > 2, Q‐value < 0.05). All proteins from CDsEVs were compared with the Vesiclepedia and Exocarta databases. GO and KEGG were performed to identify functional annotation and pathway enrichment. Proteomic profiling data have uploaded to iProx database (IPX0009882000).

### Single CDsEV Sequencing Data Analysis

The single CDsEV sequencing data analysis was performed according to a previous report by Lou et al.^[^ ^21a^ ^]^ Sequencing experiments and analyses were conducted by the Personal Biotechnology Co., Ltd., Shanghai, China. Briefly, a total of 20000 CDsEVs were used for single‐EV sequencing by 10× Genomics. The CB2 software (version 1.8.0) was used to accurately identify real EVs based on the UMI counts associated with each barcode. Subsequently, the single EV data were qualitatively controlled and normalized prior to clustering. The batch effect was corrected for by using the harmony (v 0.1.1) package to integrate single CDsEV and scRNA‐seq data for the cochlea (GSE135913) from the Gene Expression Omnibus database (http://www.ncbi.nlm.nih.gov/geo).

### Cell Culture and sEV isolation

HEK‐293T cells were cultured in DMEM (Gibco, 11995500) supplemented with 10% fetal bovine serum (FBS) (Vazyme, F101, pre‐ultracentrifuged to remove EVs), and 1% penicillin‐streptomycin antibiotics (Gibco, 15140122) in 5% CO_2_ at 37 °C. After 12 h, the FGFR1‐EGFP‐FLAG plasmid was transfected into HEK‐293T cells with Lipofectamine 2000 (Invitrogen, 11668027). The cell culture supernatant was collected after 48 h, and FGFR1‐overexpressing sEVs (FGFR1‐sEVs) were isolated using an sEV isolation kit (Umibo, UR52111) following the manufacturer's instructions.

For the cell viability test, HEI‐OC1 cells were seeded into 96‐well plates, and the FGFR1 inhibitor PD‐166866 (Selleck, S8493) at a final concentration of 0, 20, 25, 30, or 45 µM was added for 24 h. The cell viability was tested by CCK8 kit (Beyotime, C0038). For FGFR1‐sEV function tests, the experimental groups were incubated with 50 µg FGFR1‐sEVs for 12 h. Subsequently, 25 µM PD‐166866 and 50 µg FGFR1‐sEVs were added, and the cells were incubated for another 24 h. Cells treated only with or without 25 µM PD‐166866 were used as controls. After the treatment, the CCK8 kit was used to measure cell viability in all groups.

### Basilar Membrane (BM) Explant Culture

The BMs were dissected from the cochleae of wild‐type P2–3 neonatal mice under sterile conditions. Then the BMs were affixed to the slide using Cell‐Tak (Corning, 354240), cultured in DMEM/F12 (Gibco, 11330‐032) supplemented with 2% B27 (Gibco, 17504044), 1% N‐2 (Gibco, A1370701), and 50 µg ml^−1^ ampicillin (Beyotime, ST008) at 37 °C with 5% CO_2_ in four‐well dish, and allowed to recover for 12 h. BM explants were subjected to a 24 h treatment with a final concentration of 0, 15, 30, or 50 µM PD‐166866. After that, the BMs were stained by HCs marker myosin7a and SGN marker Tuj1 for analysis.

### Plasmid Construction

The *Fgfr1*(NM_010206.3) gene was cloned into the pCMV5 plasmid by Shanghai Taitool Bioscience. The FLAG tag (DYKDDDDK) and EGFP were added to the C terminus of *Fgfr1* by PCR. A detailed plasmid synthesis map is provided in Figure S4B (Supporting Information).

### Statistical Analysis

All data are presented as the mean ± standard error of the mean (SEM). Data analysis was conducted using GraphPad Prism 8. Two‐tailed, unpaired Student's *t*‐tests and one‐way ANOVA were performed to calculate the statistical significance between the different groups at three independent experiments, and statistical significance was considered when *p* < 0.05.

### Ethics Approval Statement

Every animal study adhered to the approved guidelines established by the Animal Care and Use Committee of Southeast University (20230222030) as well as the National Institutes of Health's Guide for the Care and Use of Laboratory Animals. The minimum number of animals was used, and every attempt was made to reduce the suffering of the animals.

## Conflict of Interest

The authors declare no conflict of interest.

## Author Contributions

P.J., X.M., and X.W. contributed equally to this work. S.Z. and P.J. designed the experiments and wrote the manuscript. X.M. and X.W. conducted the bioinformatics analysis. P.J., J.H., Y.W., and J.A. dissected the cochleae and performed the experiments. H.X., M.D., Y.L., X.T., B.S., W.T., and Z.Y. participated in the in vitro culture experiments. S.Z. and R.C. made revisions to the manuscript.

## Supporting information



Supporting Information

## Data Availability

The data that support the findings of this study are available from the corresponding author upon reasonable request.
