# Supplementary material for: Isolation and Comprehensive Analysis of Cochlear Tissue‐Derived Small Extracellular Vesicles
Source: Adv Sci (Weinh). 2024 Nov 5;11(48):2408964. doi: 10.1002/advs.202408964 (PMC11672270; doi:10.1002/advs.202408964)
Supplement: Supplementary file 1 — Supporting Information [file ADVS-11-2408964-s001.docx]

Supporting Information

**Isolation and Comprehensive Analysis of Cochlear Tissue-derived Small Extracellular Vesicles**

*Pei Jiang, Xiangyu Ma, Xinlin Wang, Jingyuan Huang, Yintao Wang, Jingru Ai, Hairong Xiao, Mingchen Dai, Yanqin Lin, Buwei Shao, Xujun Tang, Wei Tong, Zixuan Ye*, *Renjie Chai^*^, Shasha Zhang^*^*

**List of contents for supporting information**

1. **Supplementary text**
2. **Supplementary method**
3. **Supplementary figures**
4. **Supplementary tables**
5. **Supplementary reference**
6. **Supplementary text**

**Combined analysis of CDsEV proteomics data of and cochlear scRNA-seq data to trace the cell origin of CDsEVs**

sEVs are secreted by all kinds of cells, and the molecular composition of sEVs can reflect their cells of origin.^[1]^ Referring to previous studies,^[2]^ we attempted to determine the origins of CDsEVs by combined analysis of the CDsEV LC-MS/MS data and scRNA-seq data of the cochlea. We downloaded scRNA-seq data for the neonatal cochlea (GSE135913), organ of Corti (OC, GSE137299), and spiral ganglion neuron (SGN, GSE195500) from the GEO database. The R package Seurat was used to reclassify the scRNA-seq data, and we grouped 13 cell clusters into 9 types of cells in the cochlea, 18 cell clusters into 13 types of cells in the OC, and 10 cell clusters into 8 types of cells in SGNs (**Figure S6A-C**). The top 5 genes for each cell type are listed in **Figure S7A-C**. More than half of the genes encoding CDsEV proteins could be detected in “cluster markers” of the cochlea (**Figure S8A**), which indicated that the proteins in CDsEVs can reflect sEVs’ cell origins, which is important for future studies. However, only 26% and 10% of genes encoding sEV proteins could be detected in “cluster markers” in the OC and SGN scRNA-seq data (**Figure S8B, C**), which is probably because the OC and SGN are only part of the cochlea and our CDsEV MS data were from the whole cochlea. Therefore, we used scRNA-seq data in the neonatal cochlea (GSE135913) for the subsequent joint analysis. We calculated the sEV secretion ability of individual cochlear cells (indicated by s-EV%) based on the protein abundance observed in the LC–MS/MS data and the gene expression levels obtained from the scRNA-seq data from a previous report.^[2]^ The results showed that all cell types could secrete CDsEVs and that single SGN cells secreted more sEVs than other cells (**Figure S8D**). We also calculated the total sEV secretion abundance of each cell cluster (indicated by t-EV%), and the results revealed that the KO cell (Kölliker’s organ cell) cluster may secrete the most CDsEVs among all the cell clusters of the neonatal cochlea (**Figure S8F**), which may be because KO cells are the most abundant cells in the neonatal cochlea (indicated by cell%) (**Figure S8E**).

1. **Supplementary method**

**Combined analysis of CDsEV MS data with scRNA-seq data from the cochlea**

We downloaded scRNA-seq data of neonatal mouse cochleae (GSE135913), OC (GSE137299) and SGNs (GSE195500) from the GEO database (http://www.ncbi.nlm.nih.gov/geo). The scRNA-seq data were processed using the R package Seurat (v2.3.4). Variable genes in each sample were identified with the FindVariableFeatures program. Then, the typical Seurat clustering method was used to group all of the scRNA-seq data, and the cell clusters were located and visualized using the Find Clusters and Run TSNE programs. Using the FindAllMarkers function in the Seurat package, the Cluster Markers that were comparatively more highly expressed inside a given cluster than other clusters were selected with a criterion of Log (FC) > 0.25 and P-adj < 0.05.

The combined analysis of MS data and scRNA-seq data was performed as previously reported by Liu et al.^[2]^ Briefly, the CDsEV proteins in our MS data were looked up in the list of Cluster Markers for each cluster in the scRNA-seq data and classified as Not detected, Cluster Markers, or Non-Cluster Markers accordingly. The Cell%, s-EV%, and t-EV% were calculated as Liu et al. reported. Cell% represents the percentage of a type of cell among the total number of cells in the scRNA-seq data. s-EV% is defined as the ability of each single cell to secrete sEVs. t-EV% is defined as the ability of total cells of each cell type to secrete sEVs.

1. **Supplementary figures**

**
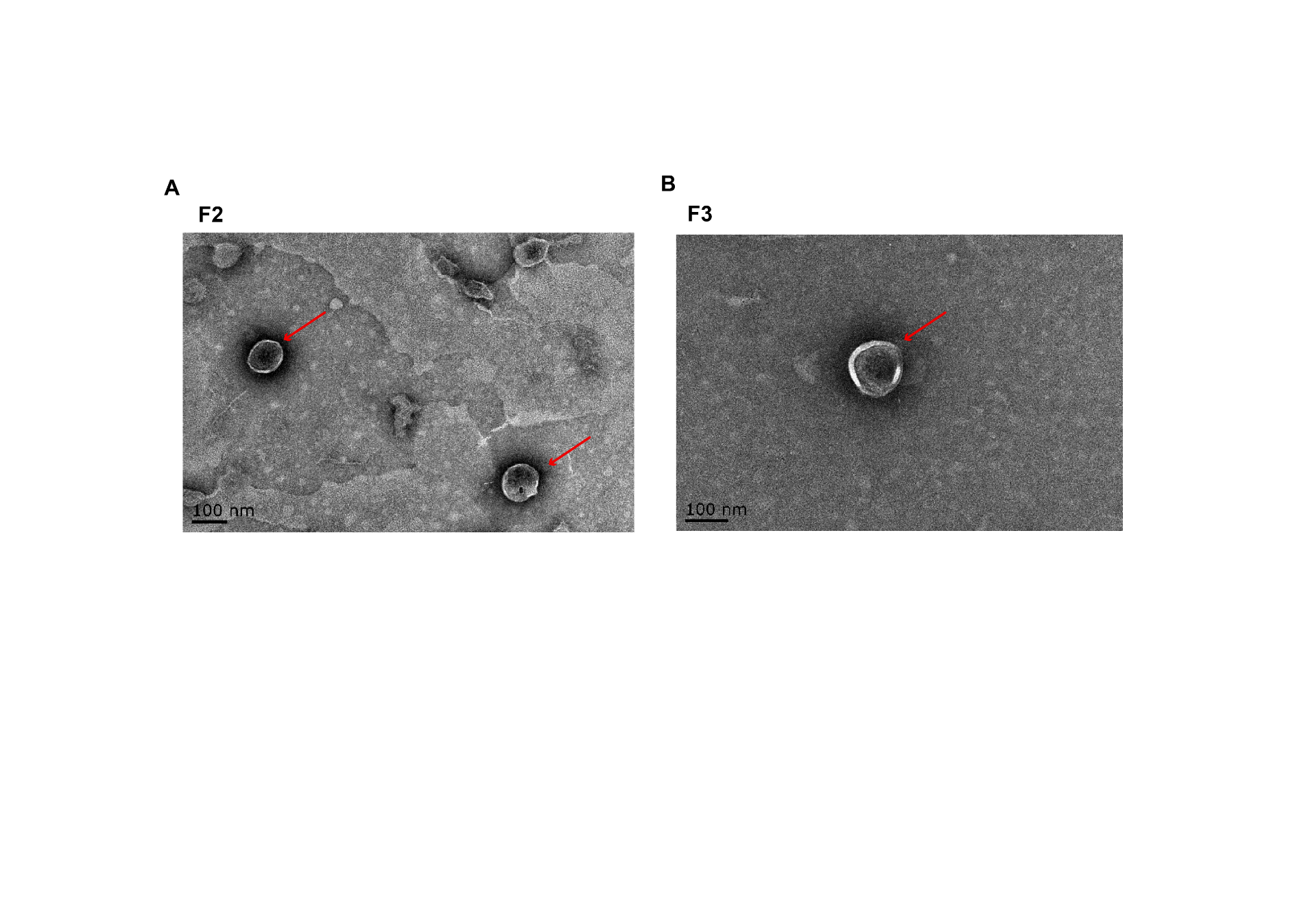
**

**Figure S1.** Characterization of particles morphology by SDGU. TEM visualization of F2 (A), and F3(B). Scale bar 100nm.


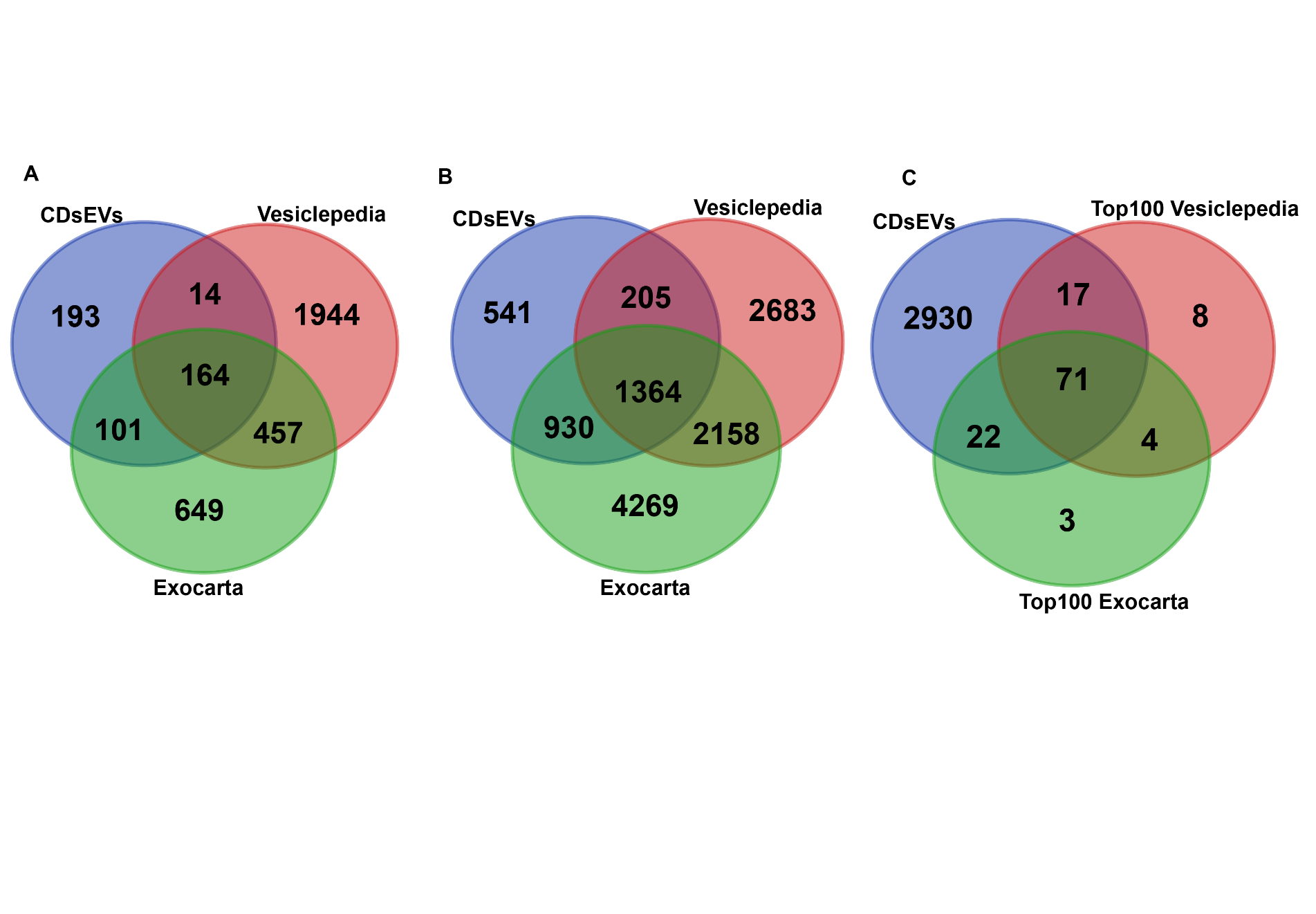


**Figure S2.** Comparison of CDsEV contents with EV databases. **(A)** Venn diagram of miRNAs in CDsEVs and the Vesiclepedia and Exocarta databases. **(B)** Venn diagram of proteins in CDsEVs and the Vesiclepedia and Exocarta databases. **(C)** Venn diagram of proteins in CDsEVs and the top 100 proteins of the Vesiclepedia and Exocarta databases.


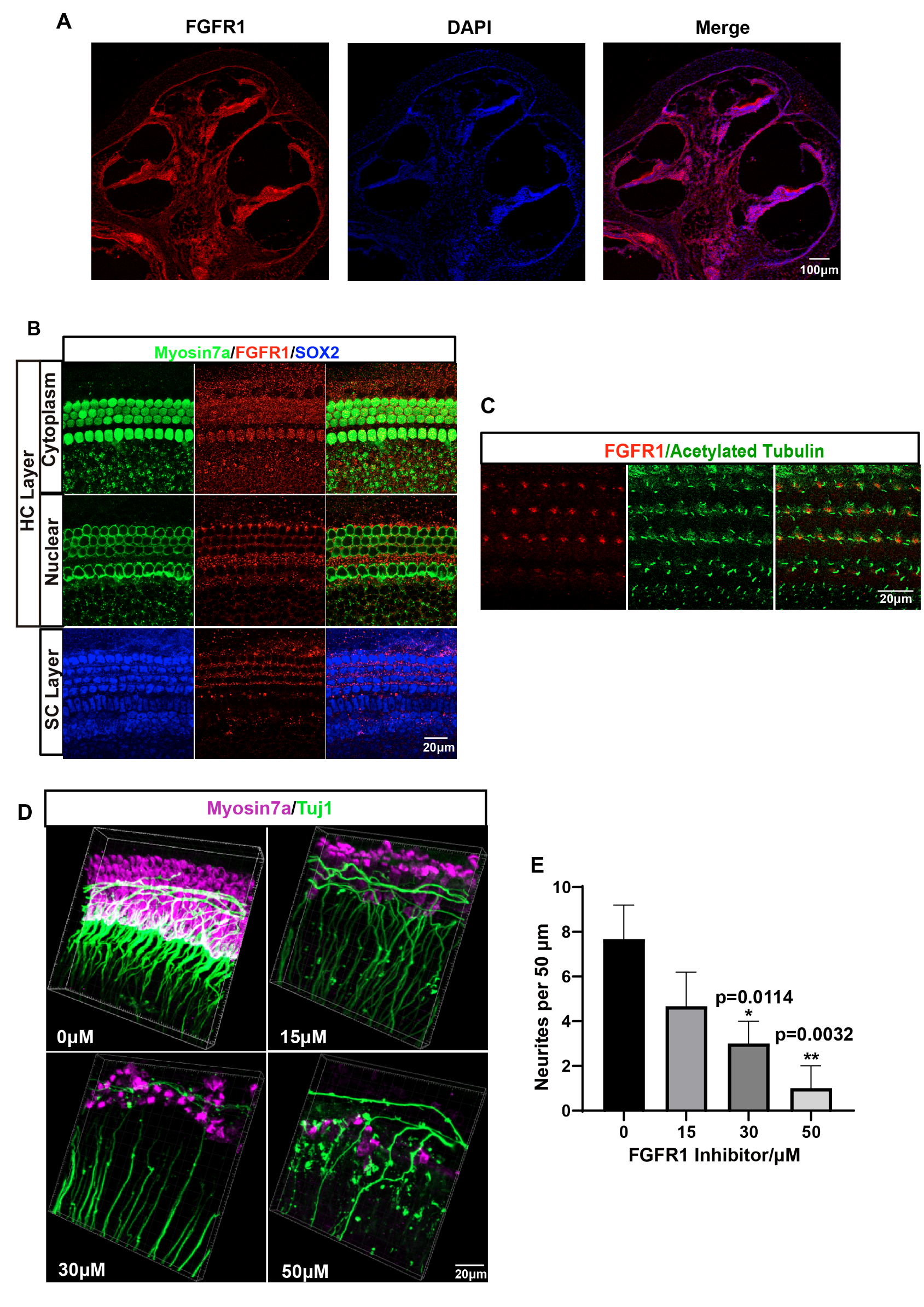


**Figure S3.** Expression of FGFR1 in the cochlea and the effect of FGFR1 inhibition on SGN. **(A-C)** Immunofluorescence staining of FGFR1 in whole cochleae (A), BM (B), and hair bundles (C) of P3 mice. DPAI (blue) in (A) labeled the nucleus. Myosin7a (green) and SOX2 (blue) in (B) were used as HC and SC markers, respectively. Acetyl-tubulin (green) in (C) was used as the kinocilium marker. Scale bar is as shown in the figure. **(D, E)** Immunofluorescence staining and 3D reconstruction (D) of BM explants treated with different concentrations (0, 15, 30, 50 µM) of the FGFR1 inhibitor PD166866 *in vitro*. Tuj1 and Myosin7a were used to label SGNs and HCs, respectively. Scale bar, 20 µm. Quantification of neurites per 50 µm of BM explants was shown in (E). * p<0.05, ** p<0,01, n=3.


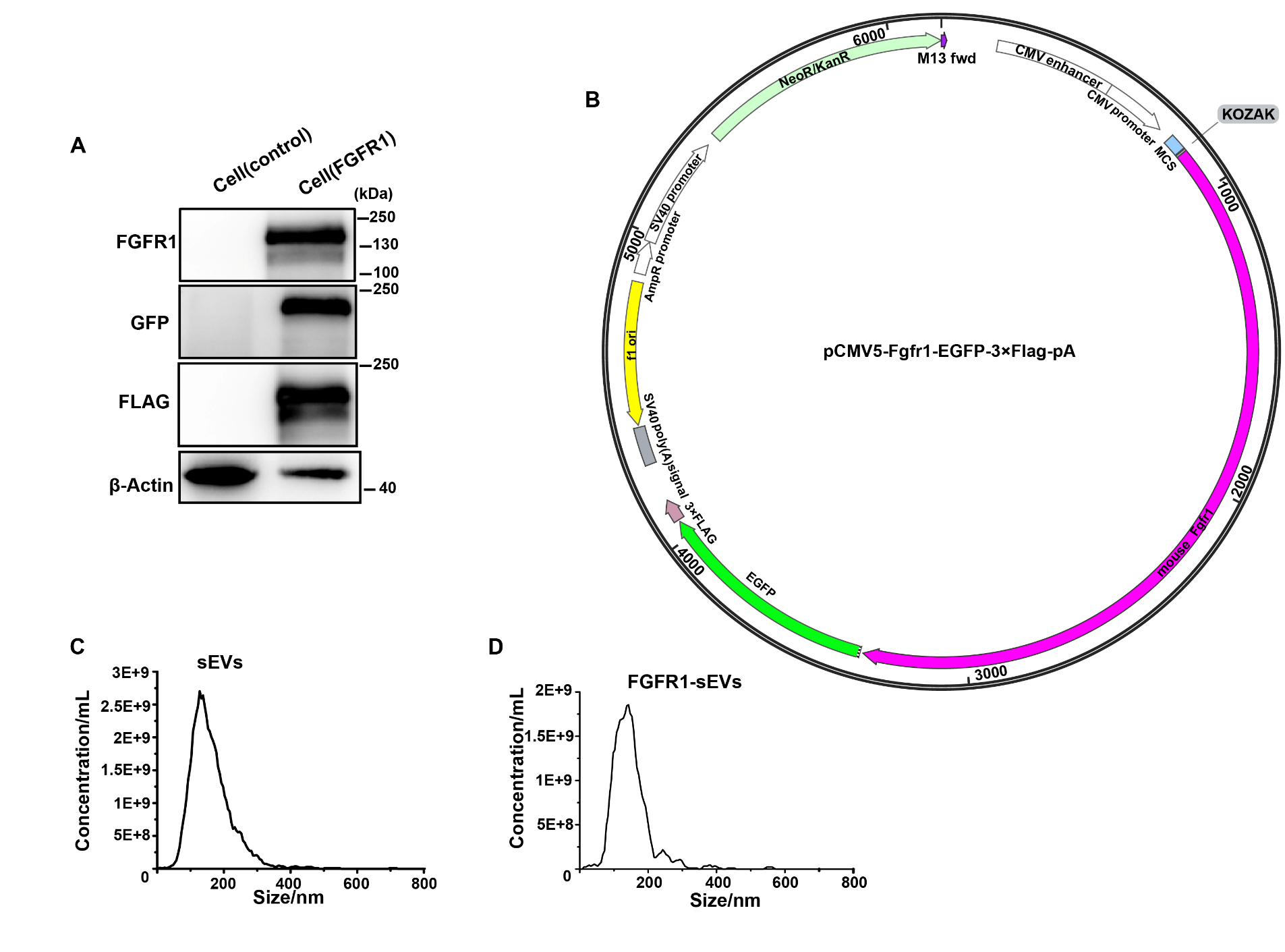


**Figure S4.** Overexpression of FGFR1 in cells. **(A)** The transfection efficiency of FGFR1-EGFP-FLAG plasmid into 293T cells was detected by WB. **(B)** The map of the FGFR1-EGFP-FLAG plasmid. **(C, D)** The concentration and size distribution of sEVs in the control group (C) and FGFR1 overexpression group (D).


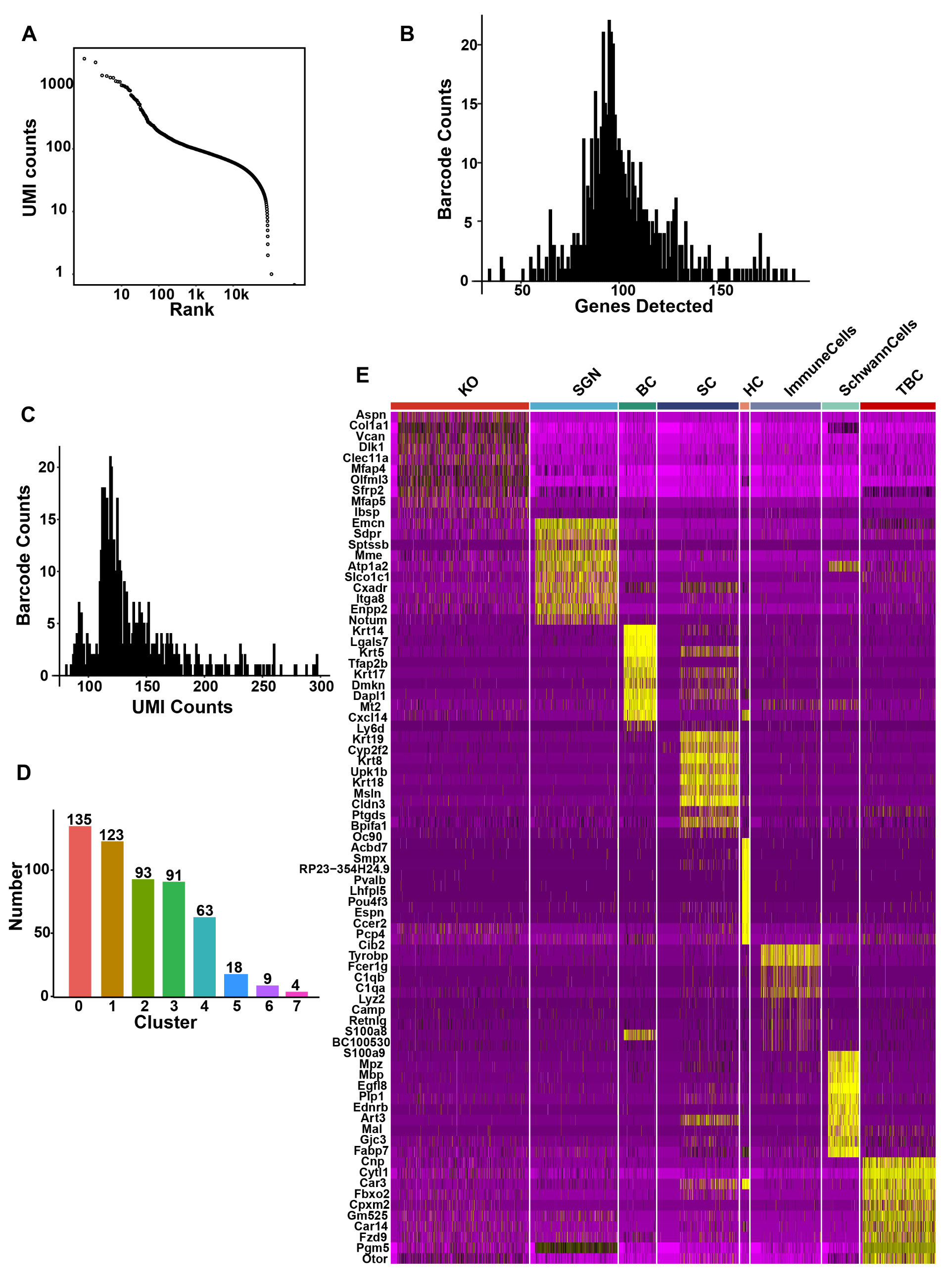


**Figure S5.** Single CDsEV-seq analysis. **(A)** Barcode rank plots showing the distribution of UMI counts associated with each barcode in the raw gene barcode matrix. **(B, C)** The distribution of the genes detected (B) and the UMI counts (C) in CDsEVs. **(D)** The number of sEVs in eight clusters of CDsEVs. **(E)** Heatmap illustrating the top five highly differentially expressed genes in each cell type in the CDsEV and cochlea integrated dataset.


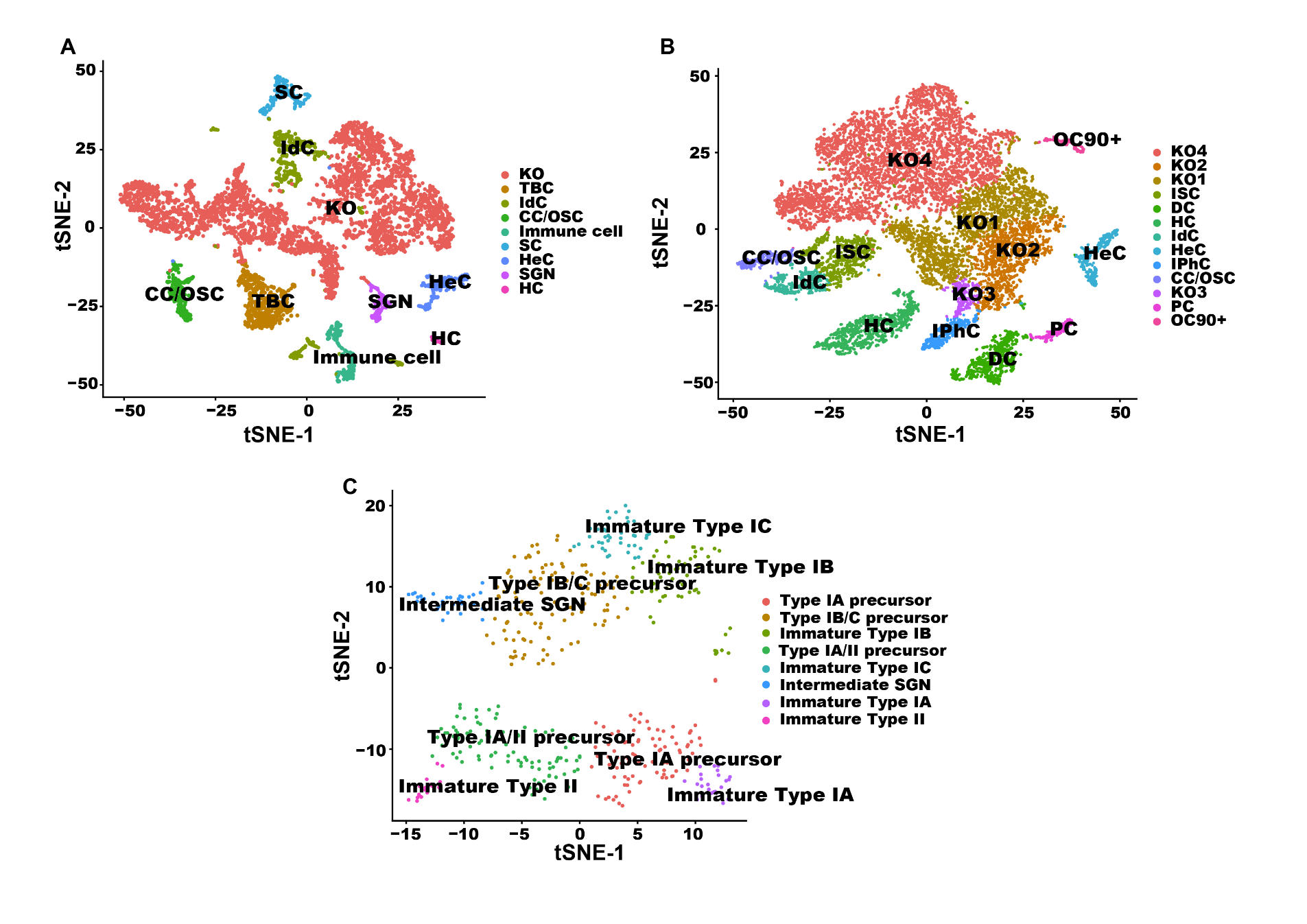


**Figure S6.** Characterization of cell types in the scRNA-seq data. **(A-C)** The tSNE plot showing cell types in the cochlea (A), OC (B), and SGN (C). Kölliker’s organ cells 1/2/3/4, KO1/2/3/4; Tympanic border cells, TBC; Interdental cells, IdC; Claudius Cells/Outer Sulcus Cells, CC/OSC; Supporting cells, SC; Hensen’s cells, HeC; Spiral ganglion neuron cells, SGN; Hair cells, HC; Deiters’ cells, DC; Inner phalangeal cells, IPhC; Inner sulcus cells, ISC; OC90 positive cells, OC90+; Pillar cells, PC.

**
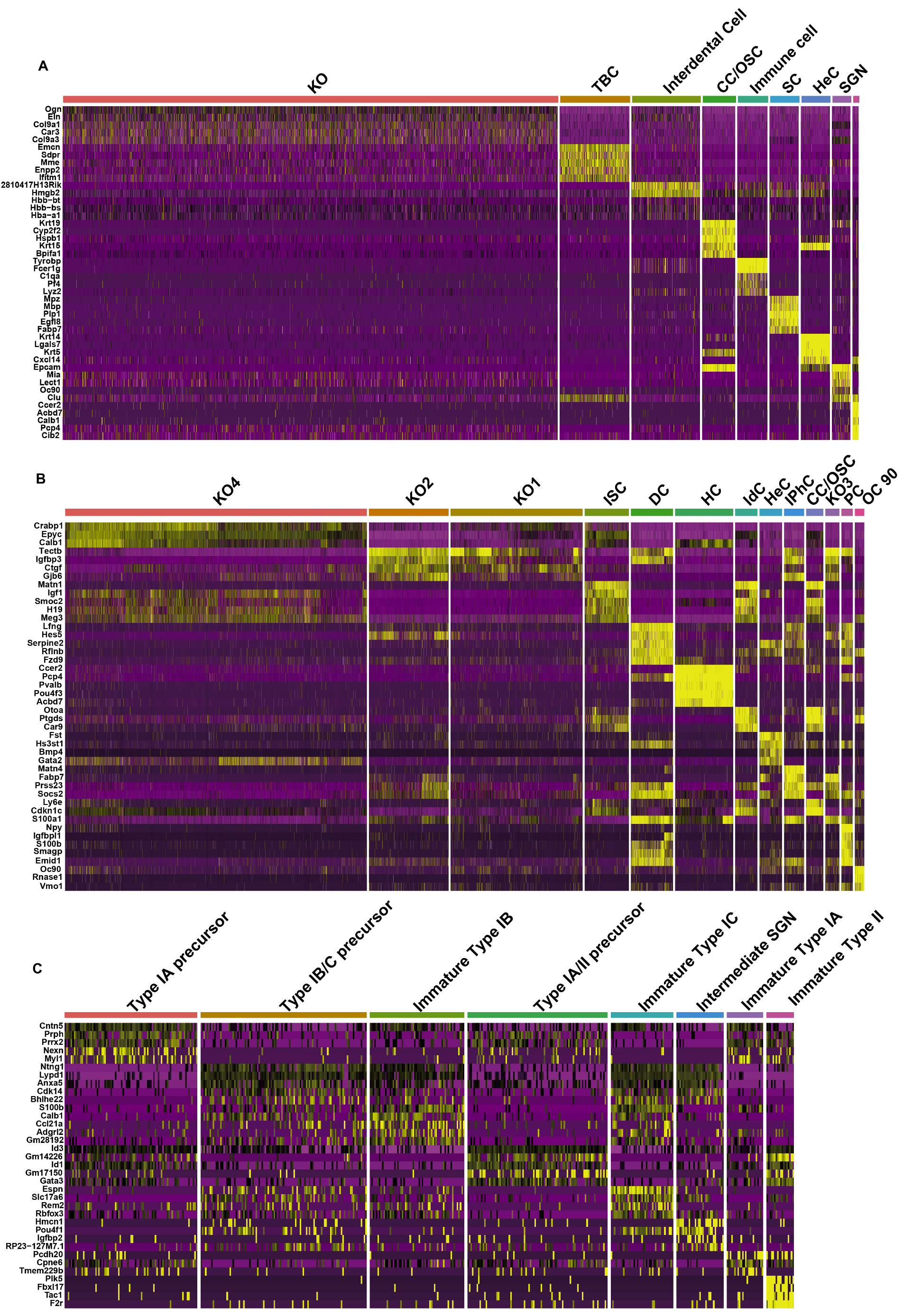
**

**Figure S7.** Heatmap of highly differentially expressed genes in each cell type. **(A-C)** Heatmap illustrating the top five highly differentially expressed genes in each cell type in the cochlea (A), OC (B), and SGN (C). Kölliker’s organ cells 1/2/3/4, KO1/2/3/4; Tympanic border cells, TBC; Interdental cells, IdC; Claudius Cells/Outer Sulcus Cells, CC/OSC; Supporting cells, SC; Hensen’s cells; HeC, Spiral ganglion neuron cells, SGN; Hair cells, HC; Deiters’ cells, DC; Inner phalangeal cells, IPhC; Inner sulcus cells, ISC; OC90 positive cells, OC90+; Pillar cells, PC.


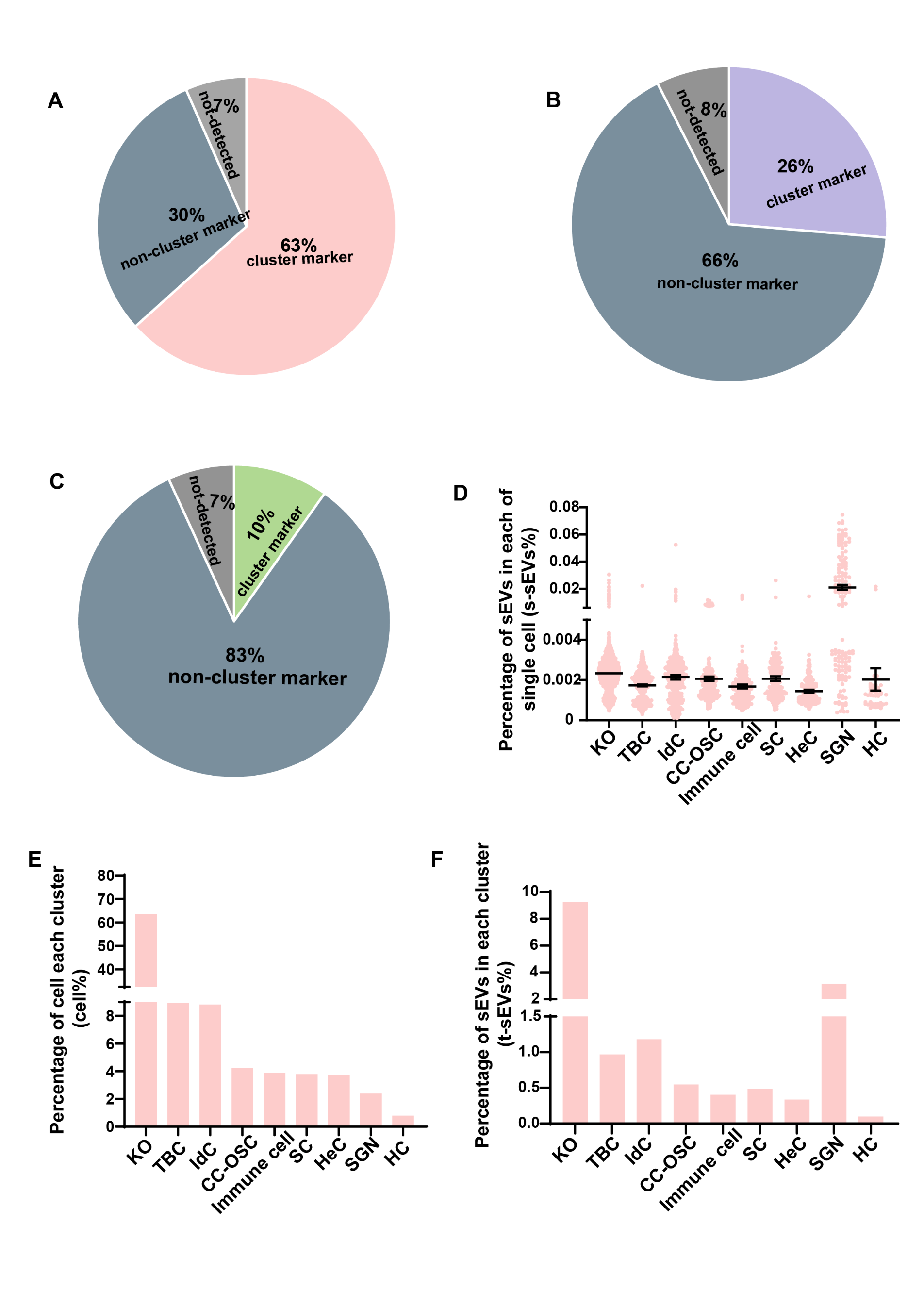


**Figure S8.** Combined analysis of CDsEV protein MS profile data with scRNA-seq data of the cochlea. **(A-C)** The proportion of “cluster marker”, “non-cluster marker”, and “not-detected” CDsEV proteins in the cochlea (A), OC (B), and SGN (C). **(D-F)** The analysis of s-sEV% (D), Cell% (E), and t-sEV% (F) of the combined analysis of CDsEV MS data and scRNA-seq data in the whole cochlea.

1. **Supplementary tables**

**Table S1 Reverse transcription PCR primer list**

| miRNA | Primer sequences (5’-3’) |
| --- | --- |
| novel miR389-3p | GTCGTATCCAGTGCAGGGTCCGAGGTATTCGCACTGGATACGACGGCTGT |
| novel miR61-3p | GTCGTATCCAGTGCAGGGTCCGAGGTATTCGCACTGGATACGACAAATCC |
| miR1291 | GTCGTATCCAGTGCAGGGTCCGAGGTATTCGCACTGGATACGACAACTGC |
| miR204-5p | GTCGTATCCAGTGCAGGGTCCGAGGTATTCGCACTGGATACGACAGGCAT |
| novel miR308-5p | GTCGTATCCAGTGCAGGGTCCGAGGTATTCGCACTGGATACGACGCTCCA |
| miR181a-5p | GTCGTATCCAGTGCAGGGTCCGAGGTATTCGCACTGGATACGACACTCAC |
| miR5121 | GTCGTATCCAGTGCAGGGTCCGAGGTATTCGCACTGGATACGACGGAGAT |
| miR27b-3p | GTCGTATCCAGTGCAGGGTCCGAGGTATTCGCACTGGATACGACGCAGAA |
| miR25-3p | GTCGTATCCAGTGCAGGGTCCGAGGTATTCGCACTGGATACGACTCAGAC |
| U6 | AACGCTTCACGAATTTGCGT |

**Table S2 Real-time Q-PCR primer list**

| miRNA | Forward (5’-3’) | Reverse (5’-3’) |
| --- | --- | --- |
| novel miR389-3p | CGGCCGAAAGCATGGGA | AGTGCAGGGTCCGAGGTATT |
| novel miR61-3p | CGCGGTGCATGGGTTT | AGTGCAGGGTCCGAGGTATT |
| miR1291 | GCGATGGCTCTTACTGAAGACTA | AGTGCAGGGTCCGAGGTATT |
| miR204-5p | CGCGTTCCCTTTGTCATCCT | AGTGCAGGGTCCGAGGTATT |
| novel miR308-5p | GCGATGGAGGACTGAGAAGG | AGTGCAGGGTCCGAGGTATT |
| miR181a-5p | CGAACATTCAACGCTGTCG | AGTGCAGGGTCCGAGGTATT |
| miR5121 | GCGCGAGCTTGTGATGAGAC | AGTGCAGGGTCCGAGGTATT |
| miR27b-3p | GCGCGTTCACAGTGGCTAAG | AGTGCAGGGTCCGAGGTATT |
| miR25b-3p | GCGCATTGCACTTGTCTCG | AGTGCAGGGTCCGAGGTATT |
| U6 | CTCGCTTCGGCAGCACA | AACGCTTCACGAATTTGCGT |

1. **Supplementary reference**

[1] H. Shao, H. Im, C. M. Castro, X. Breakefield, R. Weissleder, H. Lee, *Chem Rev* **2018**, *118* (4), 1917.

[2] B. Liu, Y. Jin, J. Yang, Y. Han, H. Shan, M. Qiu, X. Zhao, A. Liu, Y. Jin, Y. Yin, *J. Extracell. Vesicles* **2022**, *11* (5), e12223.
